# Elevated phenylacetylglutamine caused by gut dysbiosis associated with type 2 diabetes increases neutrophil extracellular traps formation and exacerbates brain infarction

**DOI:** 10.1042/CS20242943

**Published:** 2025-06-23

**Authors:** Minping Wei, Qin Huang, Fang Yu, Yun-Fang Luo, Xianjing Feng, Di Liao, Jiaxin Li, Boxin Zhang, ZeYu Liu, Jian Xia

**Affiliations:** 1 Department of Neurology, Xiangya Hospital, Central South University, Jiangxi, China; 2 Department of Neurology, Xiangya Hospital, Central South University, Changsha, Hunan, China; 3 Clinical Research Center for Cerebrovascular Disease of Hunan Province, Central South University, Changsha, Hunan, China; 4 National Clinical Research Center for Geriatric Disorders, Xiangya Hospital, Central South University, Changsha, Hunan, China

**Keywords:** gut microbiota, ischemic stroke, neutrophil extracellular traps, phenylacetylglutamine, type 2 diabetes

## Abstract

Type 2 diabetes (T2D) aggravates ischemic stroke. The association between gut microbiota-derived metabolite phenylacetylglutamine (PAGln) and ischemic stroke patients with T2D remains unclear. Therefore, we aimed to explore the change of gut microbiota and its metabolite, PAGln, in ischemic stroke patients with T2D, as well as investigate the role of PAGln in this disease. We performed two clinical cohort studies to investigate the changes of gut microbiota and PAGln in ischemic stroke patients with T2D. Then, we transplanted fecal microbiota from patients into rats and established a middle cerebral artery occlusion model. Finally, an intraperitoneal injection of PAGln was administered to rats to test whether it exacerbates brain infarction. Plasma PAGln levels were significantly higher in stroke patients with T2D compared with those without T2D. There was a positive correlation of Plasma PAGln with neutrophil extracellular traps (NETs). *Enterobacteriaceae*, *Verrucomicrobiota*, and *Klebsiella* were enriched in stroke patients with T2D and showed a significant positive correlation with PAGln levels. The rats transplanted with fecal microbes from stroke patients with T2D developed a more severe brain injury and had higher levels of plasma PAGln and NETs compared with the rats transplanted with fecal microbes from stroke patients without T2D. Additionally, rats treated with PAGln exhibited more severe brain injury accompanied by increased systemic inflammation, oxidative stress, and NET formation. Our results suggest elevated circulating PAGln levels, resulting from gut dysbiosis in stroke patients with T2D, may exacerbate brain infarction through NETs formation, systemic inflammation, and oxidative stress.

## Introduction

Stroke is a significant public health issue affecting over 10 million people annually [1]. Type 2 diabetes (T2D) is widely known to be an independent risk factor for ischemic stroke [2]. Surveys have shown that among ischemic stroke patients, up to 23.5% have diabetes mellitus, which is accompanied by higher risks of stroke recurrence, disability, and mortality [3,4]. As the global prevalence of ischemic stroke with comorbid T2D continues to rise steadily, it becomes increasingly important to identify new pathophysiological mechanisms and therapeutic targets for this condition.

A cohort study involving over 5000 individuals has revealed that patients with T2D and those who experienced adverse cardiovascular events during a three-year follow-up period exhibited elevated plasma levels of phenylacetylglutamine (PAGln) [5]. High PAGln levels have been established as independent predictors of adverse cardiovascular events [5]. PAGln is produced by the gut microbiota through the catabolism of phenylalanine, an essential amino acid, in the distal colon [6]. Recent research by Zhu et al. [7] has identified bacterial enzymes, phenylpyruvate decarboxylase (PPDC) and phenylpyruvate:ferredoxin oxidoreductase (PPFOR), capable of catalyzing the conversion of phenylalanine into phenylacetic acid. As a metabolite originating from the gut microbiota, PAGln is associated with elevated blood glucose levels and is closely related to the risk of thrombotic events, such as coronary heart disease [7,8]. Similarly, ischemic stroke represents a severe thrombotic event. Numerous studies have demonstrated alterations in the gut microbiota and its metabolites play an important role in brain injury after ischemic stroke [9,10]. For example, circulating levels of the gut microbiota-derived metabolite trimethylamine-N-oxide (TMAO) are associated with increased risk of stroke, and mice colonized with gut microbiota containing *cutC/D* suffer more severe stroke [11]. Ischemic stroke can decrease gut *Lactobacillus reuteri* and serum histidine, and exogenous histidine supplementation exerts neuroprotective activity by down-regulating inflammatory factors [12]. Gut microbiota-derived short-chain fatty acids (SCFAs) mitigate stroke severity and facilitate long-term recovery in stroke patients [13,14]. However, the relationship between gut microbiota, its metabolite PAGln, and stroke in the context of T2D remains unknown.

A separate investigation has revealed that neutrophils mediate inflammation, amplifying cerebral microvascular damage in the early stages of T2D, leading to more pronounced brain edema and nerve damage following ischemia [15]. Moreover, activated neutrophils can release neutrophil extracellular traps (NETs), intricate structures composed of DNA fibers, histones, and active components [16]. Studies have affirmed that NETs are associated with thrombosis [17,18]. Interestingly, PAGln, a metabolite produced by the gut microbiota, has also demonstrated the ability to promote thrombosis [5,19]. Increased NET formation is associated with inflammation following intestinal dysbiosis [20]. Neutrophils play a critical role as a component of the innate immune barrier in the intestinal mucosa, being the first leukocytes to be mobilized and recruited to the site of inflammation [21]. Unhealthy gut microbiota can recruit more neutrophils, increase NET production through phagocytosis and degranulation, and exacerbate the disease and systemic inflammation [22-24]. Therefore, the associations between gut microbiota, its metabolite, PAGln, and NETs in patients with stroke and T2D were investigated.

This study evaluated the gut microbiota characteristics and plasma PAGln concentration in patients with ischemic stroke, with and without T2D, through two clinical cohorts. Furthermore, the relationship between PAGln levels and NETs was analyzed. Moreover, the potential causality between gut dysbiosis, PAGln, and the worsening of stroke was investigated through fecal microbiota transplantation (FMT). Finally, PAGln intervention was given to rats to determine whether PAGln aggravates brain infarction and increases NETs formation.

## Methods

### Study population

We collected two clinical cohorts. Clinical cohort 1 included 85 patients with acute ischemic stroke (50 patients without T2D vs 35 patients with T2D). Clinical cohort 2 included 34 patients with acute ischemic stroke (19 patients without T2D vs 15 patients with T2D). All patients were from the Department of Neurology at Xiangya Hospital, Central South University (Changsha, China), between December 2019 and March 2023. The inclusion criteria were as follows: (1) aged between 18 and 80 years; (2) a first-time diagnosis of ischemic stroke, further confirmed through comprehensive neurological physical examination, head computed tomography, magnetic resonance imaging, or both; and (3) admission within two weeks of ischemic stroke onset. Moreover, patients treated with metformin or acarbose for T2D were excluded. T2D was defined based on a medical history of T2D or typical diabetes symptoms, along with random blood glucose levels ≥ 11.1 mM, fasting blood glucose ≥ 7.0 mM, or blood glucose levels, 2 hours after a glucose load ≥11.1 mM [25]. Exclusion criteria included: (1) recent use of antibiotics or probiotics within one month before admission; (2) acute inflammatory diseases, severe infectious diseases, cancer, severe liver and kidney failure, hepatic impairment, autoimmune diseases, severe mental illnesses, and gastrointestinal diseases such as inflammatory bowel disease, ulcerative colitis, and Crohn’s disease; and (3) a history of intestinal surgery. Plasma samples were collected from all participants within 24 hours, and fecal samples were obtained 48 hours after admission. The demographic and clinical characteristics of the participants in Clinical cohort 1 and Clinical cohort 2 are presented in Supplementary Tables S1 and S2, respectively.

Sample collection was conducted within the ward. Upon admission, baseline characteristics, including demographic factors and cerebrovascular risk factors such as hypertension, dyslipidemia, T2D, coronary heart disease, and smoking, were collected and documented.

Furthermore, upon admission, all patients underwent a stroke severity assessment using the National Institutes of Health Stroke Scale (NIHSS), and functional outcomes were evaluated at 90 days using the modified Rankin Scale (mRS) scores. The NIHSS score, which ranges from 0 to 42, reflects the severity of neurological deficits, with higher scores indicating more severe deficits [26]. The mRS score, ranging from 0 to 6, measures disability, with higher scores indicating more significant disability and six indicating death [27]. NIHSS and mRS scores were evaluated by two trained clinical staff members blinded to the study protocol.

### 16S rRNA amplification and sequencing of fecal microbiota samples

The fecal samples of all research subjects in clinical cohort 1 were quickly frozen in a 10 liter liquid nitrogen jar within 30 minutes of collection and then stored in a refrigerator at –80°C. Total genomic DNA was extracted from samples using a DNA kit (Magnetic Soil and Stool DNA Kit, Tiangen, DP712, Shanghai, China), following the manufacturers instructions. We selected the V3-V4 region of the 16S rRNA gene for PCR amplification and utilized specific primers, 341F (5′-CCTAYGGGRBGCASCAG-3′) and 806R (5′-GGACTACNNGGGTATCTAAT-3′). The PCR products were purified using a Qiagen Gel Extraction Kit (Qiagen, Hilden, Germany), and sequencing libraries were generated with an Illumina TruSeq® DNA PCR-Free Sample Preparation Kit (Illumina, San Diego, CA, USA). Finally, the library was sequenced on the Illumina NovaSeq platform.

Microbial data were analyzed using the Quantitative Insights into Microbial Ecology (QIIME) software package (Version 1.9.1, http://qiime.org/scripts/split_libraries_fastq.html). The effective tags of all samples were clustered using the Uparse algorithm (Uparse v7.0.1001, http://drive5.com/uparse/). Sequences were clustered into operational taxonomic units at 97% identity, and species annotations were assigned to the operational taxonomic unit sequence (with a threshold set between 0.8–1). The α and β diversity were analyzed using QIIME, and the species accumulation curve was drawn using R software (Version 2.15.3, University of Auckland, Auckland, NZ). The differences among three bacterial groups were determined using linear discriminant analysis effect (LEfSe) size with a threshold of four.

### Metagenome sequencing

Raw data from fecal samples were generated using Illumina HiSeq sequencing platforms. The raw data were filtered by quality control and removed host-derived sequences to obtain clean data. Using MEGAHIT software (v1.1.2) to assemble the clean data, and using MetaGeneMark (http://topaz.gatech.edu/GeneMark/) to predict ORFs ( ≥ 500 bp) from Scaftigs for each sample. For the ORF pretest results, use CD-HIT software to remove redundancy (http://www.bioinformatics.org/cd-hit/). The clean data of each sample were aligned to the initial gene catalog using Bowtie2. Genes with ≤2 reads in each sample were filtered out to obtain the final gene catalog (Unigenes) for subsequent analysis. Information statistics were performed based on the abundance information of each gene in each sample from the gene catalog.

DIAMOND software (https://github.com/bbuchfink/diamond/) was used to align Unigenes with sequences from the NCBI NR database (https://www.ncbi.nlm.nih.gov/) for Bacteria, Fungi, Archaea, and Viruses. The LCA algorithm was adopted (https://en.wikipedia.org/wiki/Lowest_common_ancestor) to determine the sequence of species annotation information. DIAMOND software (https://github.com/bbuchfink/diamond/) was used to compare Unigenes with the KEGG database. Anosim analysis (using the R vegan package) was used to test the differences between groups. LEfSe and Metastats analyses were used to find the differential species and genes between groups, respectively.

### Laboratory tests

Upon admission, venous blood samples were collected after a 24 hour fasting period. Subsequently, the collected samples were centrifuged at 15,000×g for 10 minutes to separate the plasma, which was then stored at –80ºC. Routine blood tests, including white blood cells, neutrophils, and lymphocytes, were performed using an automatic hematology analyzer (Hunan Desheng Medical Equipment Co., Ltd., Changsha, Hunan). Blood biochemistry parameters, such as blood urea nitrogen, serum creatinine, triglycerides, total cholesterol, low-density lipoprotein, glucose, glycosylated hemoglobin (HbA1c), and homocysteine were analyzed using an automatic biochemical analyzer (Hunan Desheng Medical Equipment Co., Ltd., Changsha, Hunan).

### Plasma PAGln measurements

Plasma PAGln concentrations in patients were quantified using targeted liquid chromatography-mass spectrometry. Briefly, 5 µl of plasma sample was mixed with 43 µl of ddH2O to obtain a tenfold diluted plasma. A 48 μl of diluted plasma was combined with 2 μl of internal standard (1 ppm D5-PAGln and 150 μL cold methanol), vortexed for 1 minute, and then centrifuged at 21,000×g for 15 minutes at 4℃. The supernatant was transferred to a clean glass bottle for testing using the AB SCIEX TripleTOF 6500 system (AB SCIEX, Foster City, CA, USA). Finally, 1 µl of supernatant was analyzed by injecting it onto an Acquity UPLC BEH C18 column (50 × 2.1 mm, 1.7 µm) at a column temperature of 40°C and with a flow rate of 0.3 ml/min. Mobile phase A contained 0.1% acetic acid in water, and mobile phase B contained 0.1% acetic acid in acetonitrile. The concentration of PAGln was measured by establishing a standard curve using concentrations of 1, 2, 10, 50, 100, and 500 ng/ml.

Plasma PAGln in rats was measured using an enzyme-linked immunosorbent assay (ELISA) kit (Jianglai, JL51250, Shanghai, China), following the manufacturer’s instructions.

### Untargeted metabolomics analysis of patient plasma samples

Plasma samples from clinical cohort 2 were analyzed by untargeted liquid chromatography coupled with tandem mass spectrometry (LC-MS/MS). Then, 100 μl of plasma was taken, mixed with 400 μl of extraction solution (MeOH:ACN, 1:1 (v/v)), which contained deuterated internal standards. The mixture was vortexed for 30 seconds, sonicated for 10 minutes in −4 water bath, incubated at −40°C for 1 hour, and centrifuged at 13,800×g for 15 minutes at 4°C. Finally, the supernatant was transferred to the injection bottle for testing. LC-MS/MS analyses were performed using an UHPLC system (Vanquish, Thermo Fisher Scientific) with a Waters ACQUITY UPLC BEH Amide (2.1 mm × 50 mm, 1.7 μm) coupled to Orbitrap Exploris 120 mass spectrometer (Orbitrap MS, Thermo). The mobile phase consisted of 25 mmol/L ammonium acetate and 25 ammonia hydroxide in water (pH = 9.75) (A) and acetonitrile (B). Sample plate temperature: 4 ℃, injection volume: 2 μl. The raw data were converted to the mzXML format using ProteoWizard. The R package and BiotreeDB (V3.0) were applied in metabolite identification.

### Quantification of NETs

Citrullinated histone H3 (H3Cit) is the most specific marker for NETs [28]. Therefore, we assessed the concentration of H3Cit to determine the levels of NETs. NETs were detected in human and rat plasma samples using a method previously described. Plasma H3Cit was measured using an enzyme-linked immunosorbent assay (ELISA) kit (Cayman Chemical, 501620, Ann Arbor, MI, USA), following the manufacturer’s instructions. Proteins were extracted from the cerebral infarction area of rats using RIPA buffer (WB3100, NCM Biotech Co., Ltd, China) supplemented with a protease and phosphatase inhibitor cocktail (P002, NCM Biotech Co., Ltd, China), and protein concentrations were determined using the BCA protein assay kit (23227, Thermo Fisher Scientific, USA). Proteins were separated via sodium dodecyl sulfide-polyacrylamide gel electrophoresis and transferred onto polyvinylidene fluoride membranes (ISEQ00010, Merck Millipore, Ireland). The latter were incubated with rabbit anti-H3Cit (1:1000, ab5103, Abcam, Cambridge, MA) and anti-H3 (1:3000, 17168–1-AP, Proteintech Group, USA) overnight at 4°C, followed by incubation with HRP-conjugated secondary antibody. The relative protein images were measured by enhanced chemiluminescence (BMU102, Abbkine Scientific Co., Ltd, China) luminous fluid. Finally, band intensities were analyzed using ImageJ software.

### Animals

Male Sprague-Dawley rats (180–230g, five to six weeks old), purchased from Hunan SJA Laboratory Animal Co. Ltd in Changsha, China, with license No. SCXK (Xiang) 2019–0004, raised at the department of laboratory animals of Central South University (Changsha, China). The animals were placed in a specific-pathogen-free environment and maintained under conditions of a 12 hour light/dark cycle, 50-55% humidity, and light intensity ranging from 100 to 200 lx. Considering the protective effect of estrogen against stroke, male rats were exclusively employed in the experimental design to control for potential confounding effects of estrogen [29]. At the end of the experiment, all animals were anesthetized with sodium pentobarbital, sacrificed by cervical dislocation, and organ tissues were removed for biochemical analysis.

### Fecal microbiota transplantation (FMT)

Before FMT, rats in both groups were given drinking water containing antibiotics (vancomycin 500 mg/L, neomycin 1 g/L, ampicillin 1 g/L, metronidazole 1 g/L (Jiangsu Jingmei Biological Technology Co., Ltd, Jiangsu, China)) for one week. Drinking water containing antibiotics was replaced every one to two days. Stool samples were randomly selected from this clinical cohort, including five stroke patients with T2D and five stroke patients without T2D. Next, five stool samples (2–3 g for each sample) from the same group were mixed in a centrifuge tube, followed by the addition of 50 ml of sterile saline. The stool homogenate was centrifuged at 12,000×g for 10 minutes, and supernatant was collected. The fecal bacterial supernatant was diluted three-fold in sterile saline to obtain approximately 150 ml. Recipient rats were orally administered 2 mL/day of the fecal supernatant at noon for one week, followed by induction of experimental stroke. Rats transplanted with fecal microbes from stroke patients with T2D were assigned to the FMT-ST group, while rats transplanted with fecal microbes from stroke patients without T2D were assigned to the FMT-SN group.

### PAGln treatment

After acclimatizing for one week, the rats were randomly divided into normal saline (NS) and PAGln groups. The rats in the PAGln group received intraperitoneal injections of 50 mg/kg PAGln every hour for three administrations. In the NS group, the rats were intraperitoneally injected with an equivalent volume of saline every hour for three administrations. The experimental stroke model was established 30 minutes following the final injection.

### Middle cerebral artery occlusion procedure

Before the middle cerebral artery occlusion (MCAO) surgery, 1 ml of orbital blood was collected from anesthetized rats (using 3% sodium pentobarbital at a dosage of 50 mg/kg, i.p., Nembutal, Ovation Pharmaceuticals Inc. Deerfield, IL, USA) to measure the PAGln concentration. The collected blood was centrifuged at 15,000×g for 10 minutes, and the plasma was separated and stored at –80°C for testing. To avoid confusion, the plasma samples collected before MCAO surgery were designated as the FMT-SN preMCAO and FMT-ST preMCAO groups, respectively. After blood collection, rats were subjected to MCAO as previously described [30]. Briefly, rats were placed in supine position, and a midline incision was made in the neck to expose both the external carotid artery (ECA) and the common carotid artery. The ECA was ligated, followed by the separation of the internal carotid artery. After cutting off the ECA, a 4–0 silicon-coated monofilament suture was inserted into the internal carotid artery. After 90 minutes of occlusion, the suture was removed, and the ECA was ligated. The neck wound was closed with sutures, and animals were allowed to recover for 24 hours before being sacrificed.

After administering anesthesia (using 3% sodium pentobarbital, 30 mg/kg, i.p., Nembutal, Ovation Pharmaceuticals Inc. Deerfield, IL, USA), blood was collected from stroke rats via cardiac puncture, and then plasma was centrifuged as previously described. The separated plasma was stored at -80°C for testing. According to the manufacturer’s instructions, we quantified plasma PAGln concentration in rats using a rat PAGln ELISA assay kit (Shanghai Jianglai Biotechnology, JL51250, Shanghai, China).

### Neurological evaluation

Neurological function of rats was assessed using the Garcia neurofunctional score 24 hours after MCAO [31]. The score includes six tests: spontaneous movement, symmetry of limb movement, symmetry of forelimb extension, climbing ability, body proprioception, and whisker response. The score ranges from 3 to 18; a lower score indicates more severe neurological deficit. The scores were evaluated by two trained experimenters who were blinded to the experimental protocol.

### Measurement of infarct volume

After anesthesia, rats were sacrificed to extract brain tissue. Coronal brain slices with a thickness of 2 mm were obtained, and five slices were cut from each brain. Staining was performed by soaking a 2% 2,3,5-triphenyltetrazolium chloride solution (Sigma, St. Louis, MO, USA) at 37°C for 30 minutes [32]. Infarct volume was calculated using Image-Pro Plus Image software (Media Cybernetics, Silver Spring, MD). Infarct volume was expressed as a percentage: infarct volume percentage = (total infarct volume/total brain volume) × 100 [33].

### Plasma TNF-α measurements

Plasma TNF-α was measured using an enzyme-linked immunosorbent assay (ELISA) kit (Thermo Fisher Scientific, 88–7340, Waltham, MA, USA), following the manufacturer’s instructions.

### Brain tissue total superoxide dismutase, glutathione peroxidase, and malondialdehyde measurements

The ischemic brain tissues of rats were mechanically homogenized under ice-water bath conditions, centrifuged at 3,000 × g for 10 min, and the supernatant was obtained. Brain tissue total superoxide dismutase (T-SOD), glutathione peroxidase (GSH-PX), and malondialdehyde (MDA) expression in brain tissue was detected by T-SOD (Servicebo, G4306, Wuhan, China), GSH-PX (Servicebo, G4310, Wuhan, China), and MDA (Servicebo, G4302, Wuhan, China) detection kits, respectively, following the manufacturer’s instructions.

### Statistical analyses

Data were statistically analyzed and managed using SPSS software (version 26.0; IBM Corp, Armonk, NY, USA) and GraphPad Prism 8.0.0 (GraphPad Software, San Diego, CA, USA). The Kolmogorov-Smirnov normality test and Shapiro-Wilk normality test were used to determine data normality. Continuous data were expressed as mean ± standard error of mean (SEM) if they followed a normal distribution, and as median and interquartile range if they deviated from normality. Categorical data were expressed as percentages. Two-sample *t*-test or Mann-Whitney *U*-test was used to assess statistical differences between two groups. Statistical differences among multiple groups were assessed by the one-way ANOVA, followed by the least significant difference test. Kruskal-Wallis H-test was used to assess statistical differences among multiple groups. Chi-square or Fisher’s exact tests were used to analyze percentages. Diversity analysis of the gut microbiota included α diversity analysis (Shannon’s index and Simpson index) and β diversity analysis (principal co-ordinates analysis [PCoA]). PCoA was performed using unweighted UniFrac distances. Analysis of molecular variance (AMOVA) was performed to assess differences in bacterial community structure between groups. Correlation was analyzed using Spearman's correlation. The ability of plasma PAGln levels to discriminate stroke patients with T2D from patients without T2D and stroke patients with good prognosis from patients with poor prognosis was evaluated using the receiver operating characteristic (ROC) analysis and the area under the ROC curve (AUC). The statistical value of *P*<0.05 was considered significant.

## Results

### Gut microbiota of stroke patients with T2D shows significant dysbiosis

**Figure 1:**
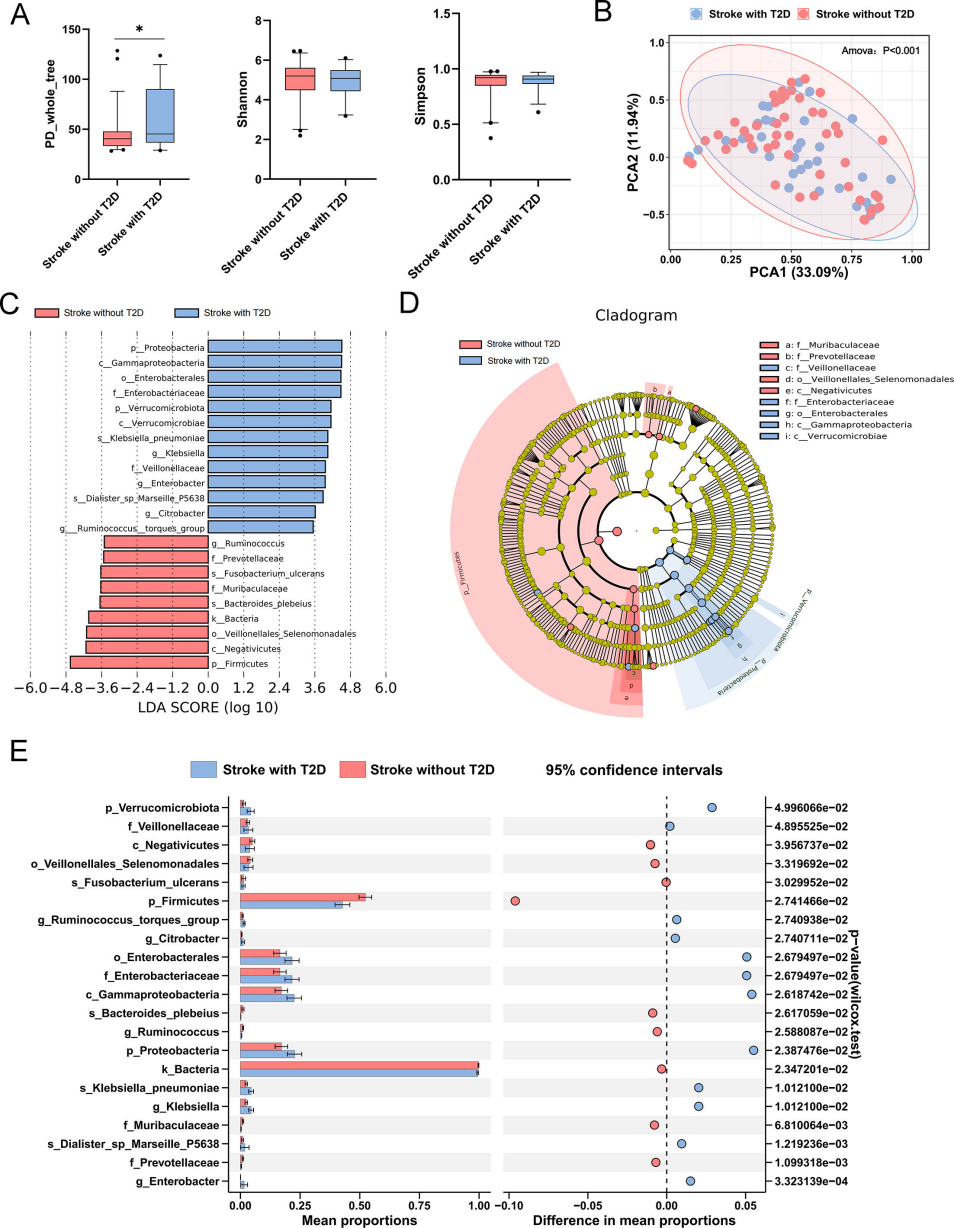
Changes in gut microbiota between two groups. (**A**) The α diversity of gut microbiota between two groups. (**B**) PCoA of β diversity based on unweighted UniFrac distances (*F* = 2.982, *P*<0.001, AMOVA). (**C**) Gut microbiota difference among three groups were evaluated using linear discriminant analysis effect size (LEfSe) (LDA score > 3.5). (**D**) The overall representation of bacteria composition in stroke with T2D and stroke without T2D groups by cladogram. (**E**) Differences in the structure of gut microbiota markers screened by LEfSe between two groups. ^*^ *P*<0.05; A, E: data were analyzed by Mann-Whitney *U*-test. T2D, type 2 diabetes.

A total of 4710 operational taxonomic units were obtained from 16S rRNA gene sequencing in 85 fecal samples, classified into 57 bacterial phyla, 417 bacterial families, and 718 bacterial genera. In comparing α-diversity of the two groups, we found that the stroke patients with T2D had higher phylogenetic diversity (PD) whole tree index compared with stroke patients without T2D, suggesting that stroke patients with T2D had a higher species diversity of gut microbiota (Figure 1A). β diversity of the stroke patients with T2D differed from the stroke patients without T2D according to the PCoA scatterplot (*P*<0.001, AMOVA; Figure 1B). LEfSe analysis was used to explore microbial markers between stroke patients with T2D and stroke patients without T2D (LDA >3.5). A total of 22 taxa were identified as potential microbial markers between the two groups (13 in the stroke with T2D group and 9 in the stroke without T2D group, Figure 1C and D). We observed a significant increase in 12 abundant taxa such as *Enterobacteriaceae, Verrucomicrobiota, Klebsiella*, and a significant decrease in 8 taxa such as *Firmicutes, Ruminococcus, Negativicutes* in the stroke with T2D group compared with the stroke without T2D group (Figure 1E). These findings suggest that stroke patients with T2D exhibit an altered gut microbiota composition, which may be the cause of stroke exacerbation by T2D.

### Gut microbiota-derived PAGln is elevated in plasma of stroke patients with T2D and positively correlated with NETs

**Figure 2:**
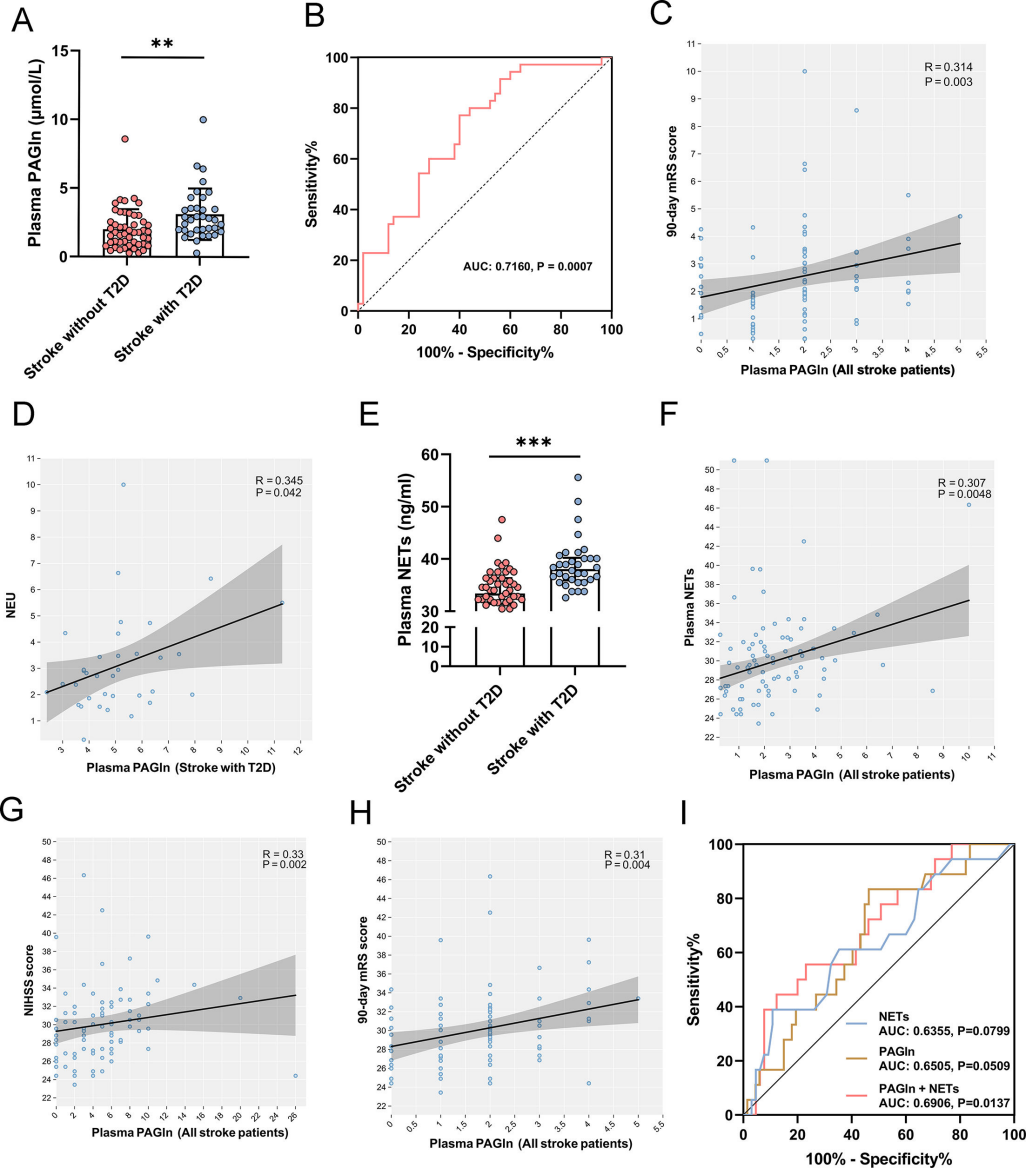
Differences in PAGln levels between the two groups and its relationship with NETs. (**A**) Plasma PAGln levels were increased in stroke patients with T2D. (**B**) Receiver operating characteristic curve analysis of plasma PAGln levels to identify stroke patients with and without T2D. (**C**) Spearman correlations between PAGln concentration and 90 day mRS score. (**D**) Spearman correlations between PAGln concentration and neutrophil. (**E**) Plasma NETs levels were increased in stroke patients with T2D. (**F**) Spearman correlations between PAGln concentration and NETs concentration. (**G-H**) Spearman correlations between admission NIHSS score, 90-day mRS score, and NETs concentration. (**I**) Receiver operating characteristic curves of plasma PAGln levels and plasma NETs levels for the identification of poor outcome prognosis (90-day mRS 3–6). ^*^ *P*<0.05, ^**^ *P*<0.01, ^***^ *P*<0.001; A, E: data were analyzed by Mann-Whitney *U*-test. NETs, neutrophil extracellular traps; PAGln, phenylacetylglutamine.

To explore the changes and clinical significance of PAGln in stroke patients with T2D, we next performed targeted metabolomics analysis of plasma samples from all stroke patients. The results of the targeted metabolomics analysis showed that the plasma levels of PAGln were significantly higher in stroke patients with comorbid T2D compared with those without T2D (*P*=0.001; Figure 2A). In addition, ROC analyses showed that plasma PAGln could distinguish stroke patients with T2D from those without T2D (AUC 0.7160, 95% CI 0.6606 to 0.7714, *P*=0.0007; Figure 2B). Next, we performed the Spearman correlation analysis. Our results showed a positive correlation between PAGln levels and 90 day mRS scores (*r* = 0.314, *P*=0.003; Figure 2C), indicating worse functional outcomes in patients with higher plasma PAGln levels. Additionally, there was a positive correlation between PAGln levels and NEU in stroke patients with T2D (*r* = 0.345, *P=*0.042; Figure 2D), indicating an association between PAGln levels and inflammation in these patients.

Therefore, we measured plasma NETs levels by evaluating H3Cit levels [28]. Our results demonstrated that plasma H3Cit levels were significantly higher in stroke patients with T2D compared with those without T2D (*P*<0.001; Figure 2E). Then, we analyzed the correlation between H3Cit levels and PAGln levels. Spearman correlation analysis revealed a significant correlation between PAGln and H3Cit levels (*r* = 0.41, *P*<0.001; Figure 2F **)**. Further correlation analysis revealed a positive correlation between plasma NETs levels and both NIHSS score upon admission (*r* = 0.33, *P*=0.002; Figure 2G) as well as 90 day mRS score post-stroke (*r* = 0.31, *P*=0.004; Figure 2H). The ROC curves demonstrated a favorable predictive capacity of the combined indices of PAGln and NETs levels for predicting poor prognosis in stroke patients (AUC 0.6906, 95% CI 0.5557 to 0.8255, *P*=0.0137; Figure 2I).

These data suggest a positive correlation between elevated plasma PAGln levels and NETs levels, which have been associated with increased stroke severity and poor prognosis at 90 days.

### Gut microbiota markers enriched in stroke patients with T2D correlate with PAGln levels

**Figure 3:**
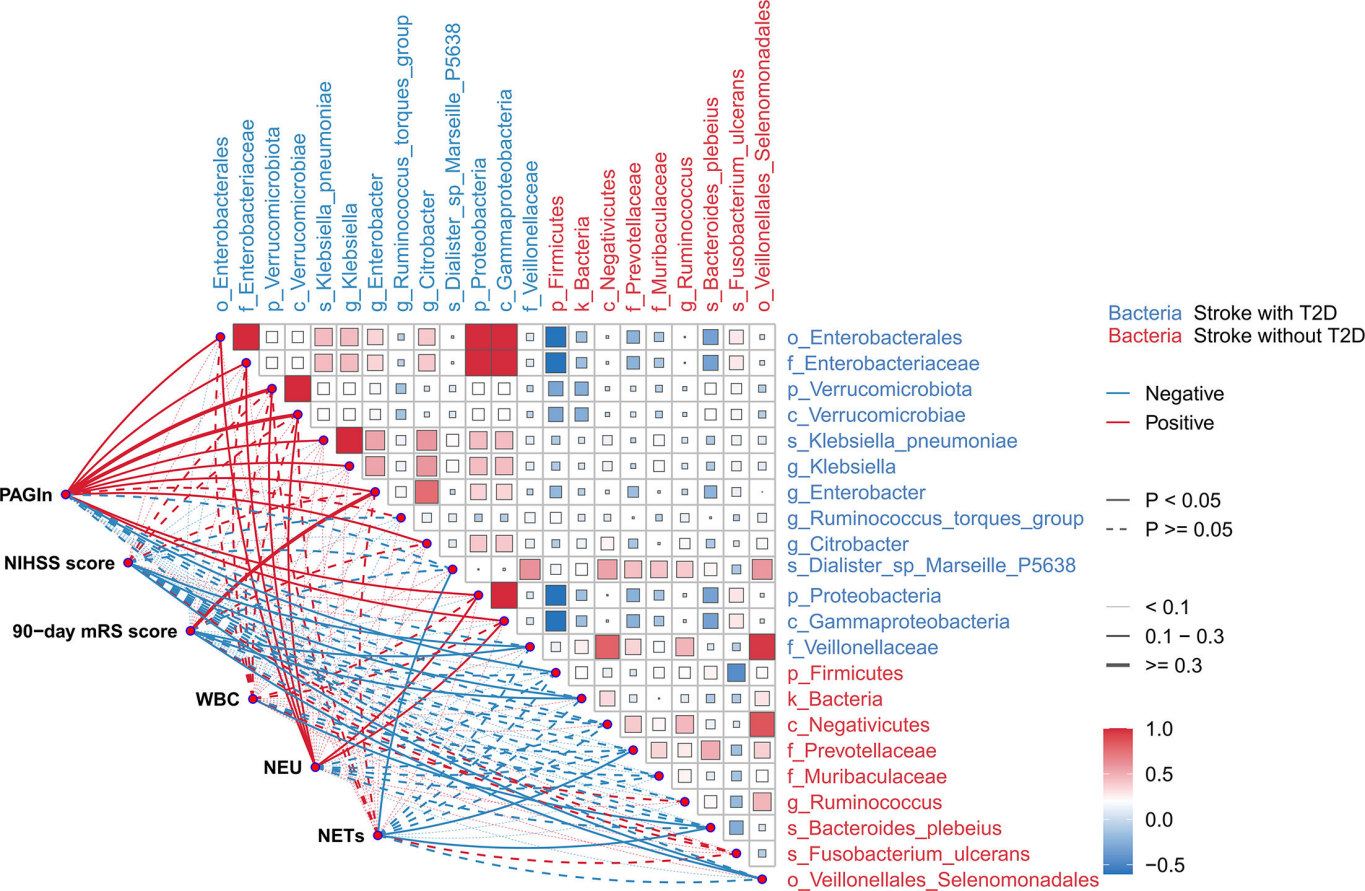
Gut microbiota markers enriched in stroke patients with T2D correlate with PAGln levels. Spearman correlations between PAGln concentration, clinical assessment, biochemical indexes, and critical gut microbiota species (LDA score > 3.5). NETs, neutrophil extracellular traps; PAGln, phenylacetylglutamine.

We performed Spearman correlation analysis to explore the association between PAGln, clinical indicators, and gut microbiota. The results showed a significant positive correlation between plasma PAGln levels and gut microbiota related in stroke patients with T2D, including *Enterobacter* (*r* = 0.293, *P*=0.007), *Verrucomicrobiota* (*r* = 0.301, *P*=0.005), *Klebsiella* (*r* = 0.228, *P*=0.036), *Citrobacter* (*r* = 0.220, *P*=0.043), *Proteobacteria* (*r* = 0.233, *P*=0.032), and *Gammaproteobacteria* (*r* = 0.229, *P*=0.035). Notably, *Enterobacteriaceae* (*r* = 0.235, *P*=0.030)*, Verrucomicrobiota* (*r* = 0.273, *P*=0.012)*, Proteobacteria* (*r* = 0.257, *P*=0.018)*,* and *Gammaproteobacteria* (*r* = 0.255, *P*=0.019) also showed a positive correlation with neutrophils (Figure 3). Additionally, the *Firmicutes*, which were reduced in stroke patients with T2D, demonstrated a negative correlation with plasma PAGln (*r* = −0.255, *P*=0.018; Figure 3). The *Enterobacter* showed a positive correlation with plasma PAGln (*r* = 0.307, *P*=0.004; Figure 3).

These findings suggest that stroke patients with T2D exhibit an altered gut microbiota composition, which may account for the elevated levels of plasma PAGln.

### Functional genes of gut microbiota in stroke patients with T2D drive the synthesis of PAGln

**Figure 4:**
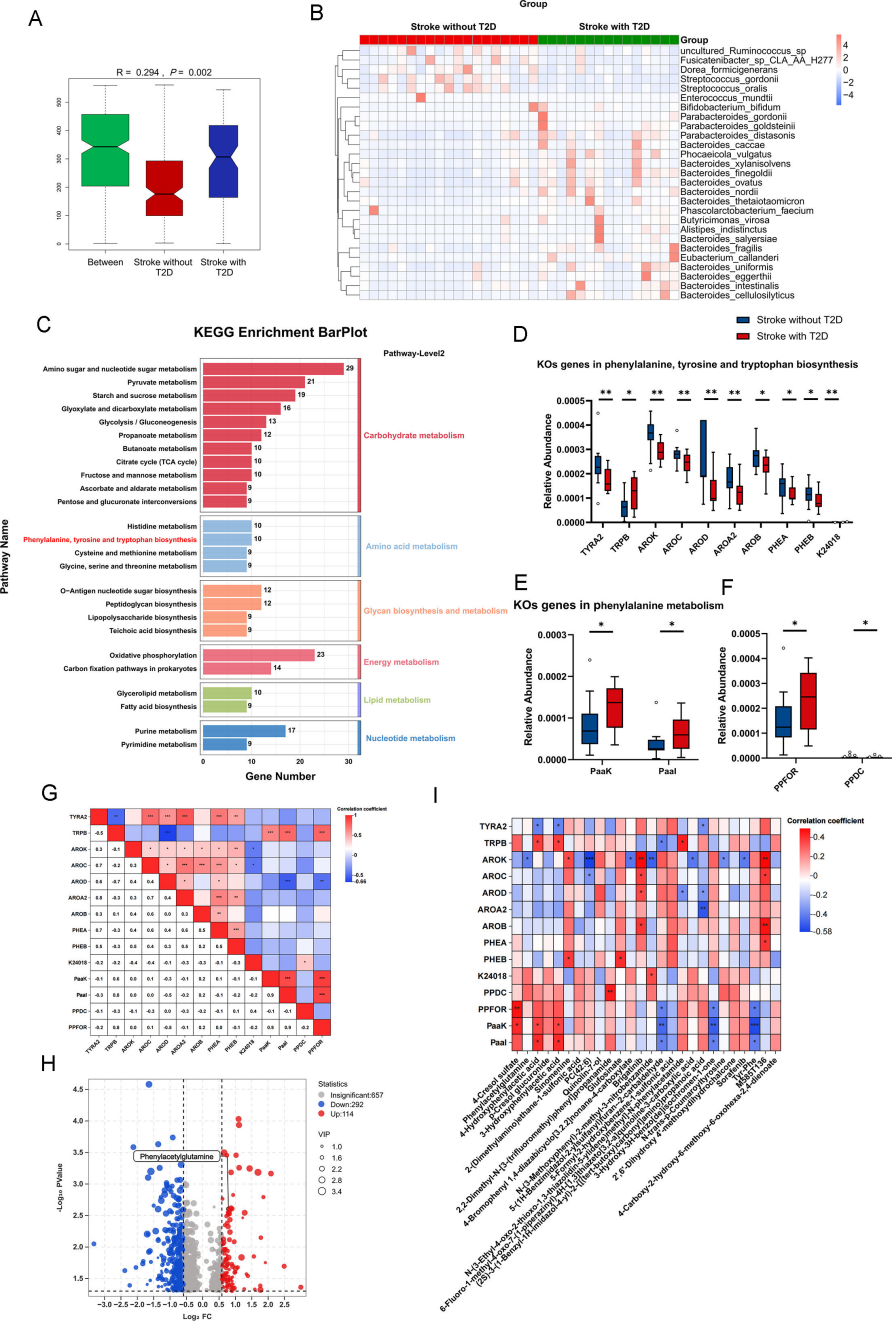
Functional genes of gut microbiota in stroke patients with T2D drive the synthesis of PAGln. **(A)** Anosim test analyzing the gut microbiota similarity between two groups of the second clinical cohort. (**B**) Heatmap of differentiated microbiota at species level by LEfSe (LDA>2.5). (**C**) KEGG enrichment analysis of differentially expressed KO genes (top 25 metabolic pathways). (**D**) Ten differentially expressed KOs genes in phenylalanine, tyrosine and tryptophan biosynthesis (MetaStat analysis). (**E**) Two differentially expressed KOs genes inphenylalanine metabolism (MetaStat analysis). (**F**) Differential analysis of relative abundance of PPFOR and PPDC (Mann-Whitney *U*-test). (**G**) Spearman correlation analysis of genes and enzymes involved in phenylalanine synthesis and metabolism. (**H**) Volcano plot of plasma differential metabolites distribution in the two groups. (**I**) Spearman correlation analysis of plasma differential metabolites with phenylalanine and metabolism-related genes and enzymes. ^*^ *P*<0.05, ^**^ *P*<0.01, ^***^ *P*<0.001. NETs, neutrophil extracellular traps; PAGln, phenylacetylglutamine.

To further validate the association between PAGln and T2D in stroke patients, we collected stool samples for metagenome sequencing and plasma samples for untargeted LC–MS/MS analysis from clinical cohort 2. Metagenome sequencing analysis revealed that samples in the clinical cohort 2 were significantly separated between different groups (*P*=0.002, Anosim test; Figure 4A). Compared with stroke patients without T2D, gut microbiota increased in stroke patients with T2D, including *Dorea_formicigenerans*, *Streptococcus_gordonii*, and *Enterococcus_mundtii* (LEfSe analysis, LDA > 2.5; Figure 4B). Moreover, there were correlations among each bacterium enriched in stroke patients without T2D, while there were fewer correlations among the bacteria enriched in stroke patients with T2D, indicating that the complexity of the gut microbiota co-occurrence network is higher in stroke patients without T2D (Supplementary Figure S1). The microbial genes were further annotated using the KEGG orthology (KO) database. MetaStat was employed to identify differentially expressed KOs between the two groups, followed by enrichment analysis of the KOs. MetaStat analysis identified 983 differential KO genes that were enriched across 658 distinct metabolic pathways, with the 25 pathways exhibiting the highest gene enrichment counts. We found the enrichment of 10 differentially expressed KO genes in the phenylalanine, tyrosine, and tryptophan biosynthesis pathway (Figure 4C). Among these 10 KO genes for phenylalanine, tyrosine, and tryptophan biosynthesis, TYRA2, AROK, AROC, AROD, AROA2, AROB, PHEA, PHEB, and K24018 were down-regulated, and TRPB was up-regulated in the gut microbiota of stroke patients with T2D (Figure 4D). In addition, in our results, two differentially expressed KO genes in the phenylalanine metabolism pathway, namely PaaK and PaaI, exhibited significantly higher expression levels in gut microbiota of stroke patients with T2D, compared with those without T2D (Figure 4E). PPFOR and PPDC, enzymes previously reported to produce phenylacetic acid, a precursor of PAGln, were also significantly elevated in the gut microbiota of stroke patients with T2D compared with stroke patients without T2D (Figure 4F). Then, we analyzed the correlation of all phenylalanine-related genes. The results showed that TRPB exhibited a negative correlation with PaaK, PaaI, and PPFOR (Figure 4G). Additionally, PPFOR demonstrated a positive correlation with both PaaK and PaaI (Figure 4G).

Differential analysis was conducted on all metabolites detected by non-targeted metabolomics, including unidentified ones, with screening thresholds of FC>1.5 or FC<0.5, VIP>1, and *P*<0.05. Our results revealed a total of 406 differentially regulated metabolites, comprising 292 down-regulated metabolites and 114 up-regulated metabolites (Figure 4H). Notably, among the screened metabolites, plasma PAGln was elevated in stroke patients with T2D (FC = 1.72, VIP = 1.73, *P*=0.0025; Figure 4H). To examine the relationship between gut microbiota and plasma PAGln in stroke patients with T2D, Spearman correlation analysis was performed to evaluate differential enriched bacteria, phenylalanine-associated KO genes, and differentially metabolites. Our results demonstrated a negative correlation between PAGln and AROK (*r* = -0.411, *P*=0.016; Figure 4I), and a positive correlation between PAGln and *Eubacterium_callanderi* (*r* = 0.348, *P*=0.044; Supplementary Figure S2).

Collectively, these findings suggest an enrichment of genes driving PAGln production in the microbial gene profile of stroke patients with T2D, which may contribute to elevated plasma PAGln levels.

### Rats receiving fecal microbes from patients with T2D and stroke displayed more severe stroke and higher levels of PAGln and NETs

**Figure 5:**
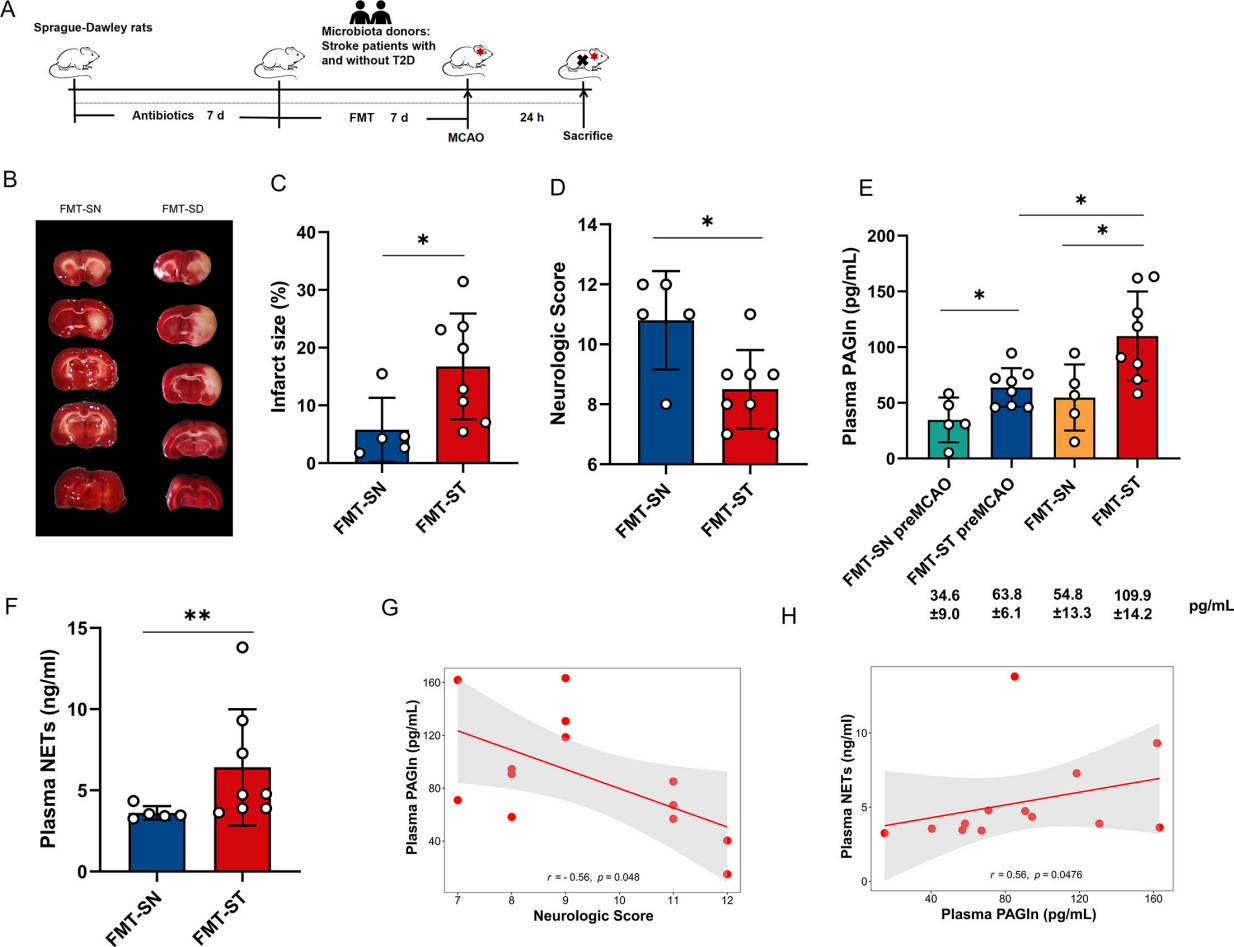
Fecal transplantation from stroke patients with T2D resulted in increased neurological impairment and plasma PAGln and NETs levels in rats. **(A)** Animal experimental design. (**B**) Representative brain slice images after 2,3,5-triphenyltetrazolium chloride staining. (**C**) Infarct volume 24 hours after experimental stroke. (**D**) Neurological function scores 24 hours after experimental stroke. (**E**) Plasma PAGln levels of rats receiving fecal microbiota transplantation. (**F**) Plasma NETs levels after experimental stroke in rats receiving fecal microbiota transplantation. (**G-H**) Spearman correlations between neurological function scores, NETs concentration and PAGln concentration in rats receiving fecal microbiota transplantation. FMT_SN (*n* = 5) and FMT_ST (*n* = 8): transplanted with fecal samples from stroke patients without T2D and stroke patients with T2D; FMT_SN_preMCAO (*n* = 5) and FMT_SD_preMCAO (*n* = 8): plasma samples collected from rats receiving fecal microbiota transplantation before experimental stroke. Data are expressed as the mean ± SEM. ^*^ *P*<0.05, ^**^ *P*<0.01 (Mann-Whitney *U*-test). NETs, neutrophil extracellular traps; PAGln, phenylacetylglutamine; T2D, type 2 diabetes.

To investigate the potential contribution of gut microbiota in stroke patients with T2D to elevated plasma PAGln levels, we treated antibiotic-treated rats with fecal transplants from stroke patients with and without T2D (donors in both groups had similar age, sex, previous history, and other baseline characteristics; see Supplementary Table S2 for details) **(** Figure 5A **)**. After 24 hours of establishing the MCAO model, rats receiving fecal microbes from stroke patients with T2D exhibited a more severe stroke compared with those receiving fecal bacteria from stroke patients without T2D, as evidenced by increased infarct volume (*P*=0.042; Figure 5B and C **)** and lower Garcia neurofunctional scores (*P*=0.042; Figure 5D **)**. Results demonstrated that prior to establishing the MCAO model, rats receiving fecal microbes from stroke patients with T2D exhibited a significant and nearly twofold increase in plasma PAGln levels compared with rats receiving fecal microbes from stroke patients without T2D (63.8 ± 6.1 vs 34.6 ± 9.0 pg/mL, *P*=0.02; Figure 5E **)**. Meanwhile, after establishing the MCAO model, plasma PAGln levels in rats receiving fecal microbes from stroke patients with T2D were also observed to be approximately twice as high as those receiving fecal microbes from stroke patients without T2D (109.9 ± 14.2 vs 54.8 ± 13.3 pg/ml, *P*=0.03; Figure 5E **)**. Notably, the PAGln levels significantly increased after inducing the MCAO model in rats receiving fecal microbes from stroke patients with T2D (63.8 ± 6.1 vs 109.9 ± 14.2 pg/ml, *P*=0.021), while the PAGln levels showed a non-significant increasing trend after inducing the MCAO model in rats receiving fecal microbes from stroke patients without T2D (34.6 ± 9.0 vs 54.8 ± 13.3 pg/ml, *P*=0.31; Figure 5E).

Given the positive correlation between PAGln levels and NETs levels in the clinical cohort, we measured the plasma NETs levels in rats. The stroke rats receiving fecal microbes from stroke patients with T2D showed significantly higher plasma NETs levels compared with those receiving fecal microbes from stroke patients without T2D (*P*=0.011; Figure 5F). Consistent with the results in the clinical cohort, Spearman correlation analysis revealed a positive correlation between levels of PAGln and NETs in rats (*r* = 0.56, *P*=0.0467; Figure 5H). Moreover, the correlation analysis revealed a negative association between PAGln and Garcia neurological function score, suggesting that higher levels of PAGln are associated with more severe stroke (*r* = -0.56, *P*=0.0480; Figure 5G **)**.

These findings suggest that gut microbiota of stroke patients with T2D contributes to elevated levels of plasma PAGln levels and NETs, as well as a more severe stroke.

### Elevated plasma PAGln aggravates brain infarction through increasing NETs formation, systemic inflammation, and oxidative stress of brain tissues

**Figure 6:**
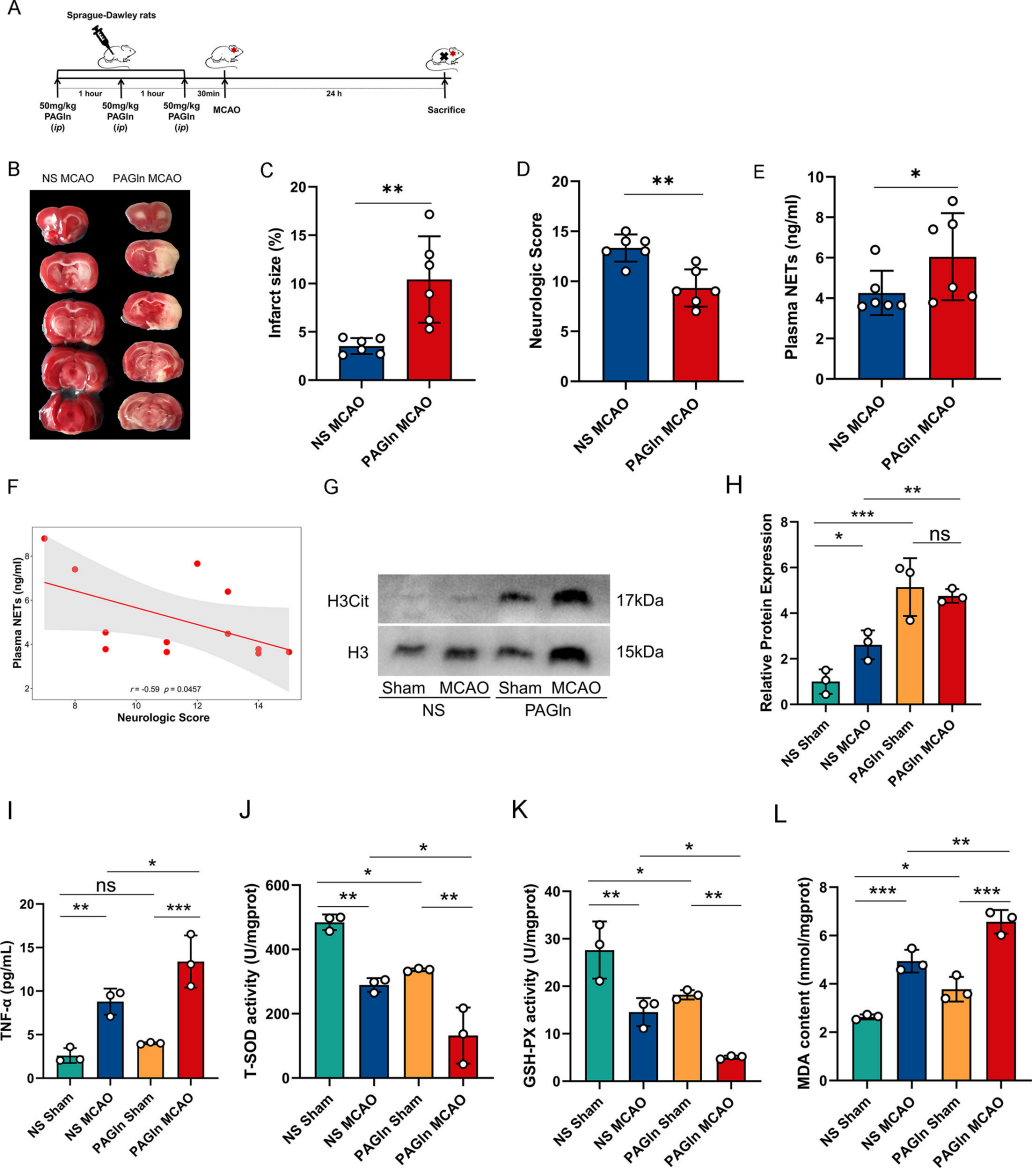
Rats treated with PAGln exhibited more severe stroke and increased NETs formation in brain tissue. (**A**) Animal experimental design. (**B**) Representative brain slice images after 2,3,5-triphenyltetrazolium chloride staining. (**C**) Infarct volume 24 hours after experimental stroke (*n* = 6/group). (**D**) Neurological function scores 24 hours after experimental stroke (*n* = 6/group). (**E**) Plasma NETs levels after experimental stroke (*n* = 6/group). (**F**) Spearman correlations between neurological function scores and NETs concentration in rats. (**G-H**) Expression of NETs in areas of cerebral infarction of rats was assessed with Western blot (*n* = 3/group). (**J**) Plasma TNF-α concentration in rats. (**J-L**) T-SOD, GSH-PX, and MAD concentration in the rats' brain tissue. Data are expressed as the mean ± SEM. ^*^ *P*<0.05, ^**^ *P*<0.01, ^***^ *P*<0.001 (Mann-Whitney *U*-test or ANOVA analysis). GSH-PX, glutathione peroxidase; MDA, malondialdehyde; NETs, neutrophil extracellular traps; PAGln, phenylacetylglutamine; T-SOD, total superoxide dismutase.

To further investigate the impact of PAGln on stroke, rats were administered intraperitoneal injections of PAGln, followed by the induction of ischemic stroke (Figure 6A). Our results showed that rats of the PAGln group revealed a significant increase in infarct volume (*P*=0.008; Figure 6B and C) and a significant decrease in Garcia’s neurofunctional scores (*P*=0.004; Figure 6D) compared with the rats of the NS group. Furthermore, plasma NET levels were quantified in rats after experiencing cerebral infarction. Results indicate that the PAGln group exhibited elevated levels of plasma NETs in comparison with the NS group (*P*=0.041; Figure 6E). Moreover, correlation analysis revealed a negative association between NETs and Garcia neurological function scores (*r* = −0.59, *P*=0.0457; Figure 6F). Subsequently, we examined the expression level of NETs in rat cerebral infarction tissue by Western blot. The results showed that the expression of NETs in rat brain tissue increased after PAGln intervention (*P*<0.0001, Figure 6G and H). Compared with the stroke rats of the NS group, NETs expression increased in cerebral infarction tissue in rats of the PAGln group (*P*=0.009, Figure 6G and H). In addition, the plasma TNF-α concentration in PAGln-treated MCAO rats was significantly higher than that in saline-treated MCAO rats (*P*<0.05, Figure 6I). To evaluate oxidative stress in rats with cerebral infarction after PAGln intervention, we detected oxidative stress-related indicators. In contrast to the NS MCAO group, the levels of T-SOD and GSH-PX were obviously reduced, and the levels of MAD were obviously enhanced in the PAGln MCAO group (*P*<0.05, Figure 6J–L).

These findings suggest that PAGln may exacerbate brain infarction via the formation of NETs, systemic inflammation, and oxidative stress in brain tissues.

## Discussion

**Figure 7:**
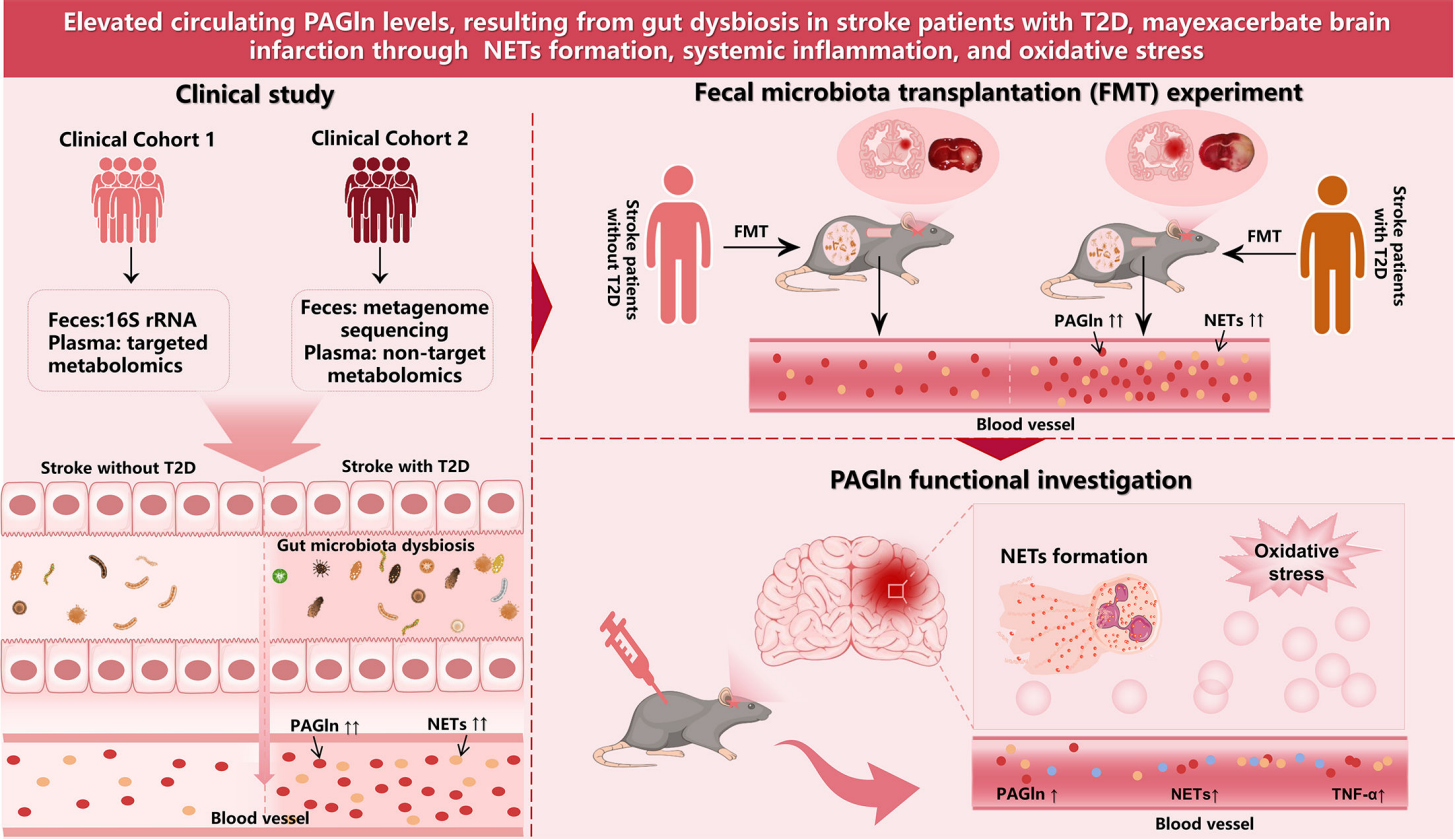
Ischemic stroke patients with T2D exhibit disruptions in gut microbiota and elevated plasma PAGln levels, and rats receiving fecal microbes from patients with T2D and stroke displayed more severe stroke and higher levels of PAGln and NETs. Elevated PAGln levels may exacerbate brain infarction through NETs formation, systemic inflammation, and oxidative stress. NETs, neutrophil extracellular traps; PAGln, phenylacetylglutamine; T2D, type 2 diabetes.

This is the first study to explore the association between the gut microbiome-derived metabolite PAGln and the brain in the context of ischemic stroke with coexisting T2D. Our data suggest that (i) significant dysbiosis in the gut microbiota of patients with stroke and T2D, leading to increased plasma PAGln levels, and (ii) elevated PAGln levels can worsen brain infarction, correlating with NETs formation, systemic inflammation, and oxidative stress (Figure 7).

Our results demonstrate elevated plasma PAGln levels among patients with stroke, with even higher levels observed in those with comorbid T2D. Plasma PAGln is cleared by the kidneys. Therefore, impaired renal function, leading to reduced PAGln plasma clearance, contributes to elevated PAGln circulating levels [34,35]. Our results reveal no significant differences in blood urea nitrogen or serum creatinine levels between those patients with stroke, with and without T2D, indicating that T2D primarily influences increased plasma PAGln production in patients with stroke and comorbid T2D. Tang et al. [36] found that plasma PAGln levels were significantly higher in patients with heart failure and diabetes mellitus than in those with heart failure alone, suggesting a potential association between elevated plasma PAGln and diabetes mellitus. Research has demonstrated that metabolic disorders such as insulin resistance, dyslipidemia, and elevated fatty acids disrupt the nitric oxide-mediated signaling pathway, leading to increased platelet activity, aggregation, and the development of thrombosis and atherosclerotic lesions [37]. Furthermore, there is evidence suggesting that PAGln can enhance platelet invasiveness [5,19]. . Therefore, our study suggests that increased circulating PAGln levels may mediate the involvement of diabetes in platelet activation and contribute to the pathogenesis of diabetes-related thrombotic diseases. In the future, targeting PAGln could be a potential therapeutic approach for improving diabetes-related thrombotic diseases. However, the molecular mechanisms through which PAGln promotes thrombosis and exacerbates cerebral infarction in diabetes require further investigation.

PAGln is a metabolite produced by the gut microbiota during phenylalanine degradation. In the large intestine, phenylalanine is metabolized by gut microbiota into phenylacetic acid, which then enters the liver and is further metabolized into PAGln. Our study identified disrupted gut microbiota in patients with stroke and T2D, characterized by an increased presence of PAGln-associated gut microbiota, including *Enterobacteriaceae, Verrucomicrobiota,* and *Klebsiella*. Previous research has reported an association between stroke mortality and *Enterobacteriaceae*, as they exacerbate systemic inflammation and cerebral infarction by up-regulating inflammatory factors such as tumor necrotic factor-α and interleukin-1β through the lipopolysaccharide-Toll-like receptor-4 pathway [10]. Moreover, research has indicated that *Proteobacteria*, a bacterial phylum that includes *Enterobacteriaceae*, possesses the PPDC gene responsible for converting phenylalanine to phenylacetic acid, an intermediate metabolite in the production of PAGln from phenylalanine [7,19]. Our findings suggest that *Enterobacteriaceae*, positively correlated with PAGln, may contribute to the exacerbation of brain injury in patients with T2D and stroke driven by diabetic conditions. In our study, *Akkermansia* (99.4%) dominated the *Verrucomicrobiota. Akkermansia* is conventionally recognized for its potential to support intestinal epithelial health, thereby enhancing intestinal barrier function, producing beneficial short-chain fatty acids, and improving metabolic disorders [38,39]. However, its role in the development of stroke and diabetes mellitus remains unclear. Previous studies have reported elevated levels of *Akkermansia* in individuals with prediabetes and diabetes, and in diabetic animal models [40,41]. Furthermore, an increase in *Akkermansia* has been associated with mucous layer erosion and enhanced pathogen filtration in the intestinal epithelial layer when dietary fiber is lacking in the host’s gut [42,43]. In our investigation, *Verrucomicrobiota* was enriched in patients with stroke and T2D, and this increase showed a significant positive correlation with PAGln levels. Therefore, T2D was hypothesized to increase the abundance of specific *Akkermansia* species associated with PAGln metabolism. Alternatively, under T2D conditions, *Akkermansia* may exhibit enhanced enzyme activity involved in PAGln metabolism, ultimately resulting in elevated PAGln levels in the host’s plasma. Therefore, further investigation of the role played by different *Akkermansia* species within the context of stroke and concurrent T2D is imperative for future research.

PaaK, named phenylacetate-CoA ligase, the initial enzyme in the metabolism of phenylacetate, is involved in the degradation of the aromatic amino acid phenylalanine [44,45]. PaaI, named acyl-CoA thioesterase, acting on hydroxylated phenylacetyl-CoA substrates, is involved in phenylacetic acid degradation [44,46]. PPFOR transforms phenylpyruvic acid, the next level metabolite of phenylalanine, into phenylacetic acid through an oxidative process [7]. Our findings demonstrated that PPFOR, PaaK, and PaaI were significantly up-regulated in the gut microbiota of stroke patients with T2D. Notably, both PaaK and PaaI exhibited positive correlations with PPFOR. These results indicate the pathway for PAGln production in the intestine of stroke patients with T2D is highly active, which may contribute to the elevated plasma PAGln levels observed in these patients. TRPB, named tryptophan synthase beta chain, is the enzyme that synthesizes tryptophan. Tryptophan is synthesized through the shikimate pathway along with other aromatic amino acids, phenylalanine and tyrosine [47]. Our results demonstrated that TRPB was significantly elevated in the gut microbiota of stroke patients with T2D and exhibited a positive correlation with phenylalanine metabolism-related genes. This suggests the potential for synergistic expression and interaction between genes involved in phenylalanine catabolism and tryptophan synthesis within the gut microbiota.

Our results highlight a significant correlation between PAGln and *Klebsiella*, a bacterium possessing the PPDC gene responsible for converting phenylalanine into phenylacetic acid [7,19,40]. Consequently, we speculate that the PPDC-mediated pathway is postulated to predominantly contribute to the elevated PAGln levels observed in patients with stroke and T2D. Future studies should focus on validating the abundance of PPDC and PPFOR in the gut microbiota of these patients.

FMT currently stands as a prevalent intervention strategy for investigating the causal relationship between gut microbiota and diseases, and a promising treatment method that has undergone extensive research [48]. Although species differences lead to variations in the gut microbiome between humans and rodents, numerous studies have substantiated that the gut microbiota community structures and clinical phenotypes in rodents can resemble those of human donors after receiving fecal microbiota transplants [49-51]. This enables us to study the function of human gut bacteria in animals more effectively. In this research, the fecal microbiota of patients with stroke and T2D was transplanted into rodents, resulting in increased infarct volume and worsened neurological dysfunction. These results indirectly support the potential causal relationship between gut microbiota disorder and poor stroke prognosis, suggesting that T2D may exacerbate stroke brain injury through gut microbiota disorder. Singh et al. [52] demonstrated that animals with severe stroke exhibited more pronounced dysbiosis in their gut microbiota compared with those with mild stroke. In our findings, animals receiving fecal microbiota from patients with stroke and T2D displayed worse stroke outcomes and higher plasma PAGln levels, indicating an association between elevated plasma PAGln and gut microbiota dysbiosis. This observation provides direct evidence of increased PAGln levels in patients with T2D and stroke, partially attributed to disruptions in intestinal microorganisms. Our results further indicated increased post-stroke PAGln levels in rats receiving fecal microbes from patients with stroke and T2D. Therefore, acute stroke stress is speculated to potentially enhance the growth and proliferation of PAGln-producing bacteria, contributing to elevated PAGln levels. Our results revealed a negative correlation between the neurological score and plasma PAGln levels in rats receiving FMT. When rats were administered PAGln, those that had experienced a stroke exhibited worsened neurological dysfunction and an increased infarct volume. In summary, targeting PAGln may represent an effective therapy for improving brain injury in patients with T2D and stroke. PAGln, a metabolic product of gut microbiota, can be reduced by remodeling the gut microecology through the FMT intervention strategy. This approach may potentially improve brain injury in patients with T2D and stroke. Identifying strains related to PAGln metabolism and targeting bacteria responsible for PAGln production should be further investigated to enhance the prognosis of patients with stroke and T2D.

Consistent with previous research, our findings revealed elevated NETs levels in patients with stroke, with a further increase observed in those with coexisting T2D. In the arterial microenvironment of thrombosis, activated platelets trigger neutrophils to generate and release NETs containing thrombotic tissue factor through various mechanisms, including neutrophil autophagy induced by high-mobility group box-1 protein expression, inorganic polyphosphate as an inducer for NETs, and activation of platelet Toll-like receptor 4 [53-56]. Meanwhile, NETs can exacerbate thromboinflammation by promoting thrombin and fibrin formation and binding to platelet-derived microparticles and coagulation factors [57]. Our data demonstrates a positive correlation between plasma PAGln levels and NETs. In animal experiments, rats receiving fecal microbiota from patients with T2D and stroke exhibited elevated levels of both PAGln and NETs, with a positive correlation between these two variables. In addition, we observed that rats treated with PAGln not only exhibited enhanced NETs formation but also demonstrated a significant increase in inflammatory response and oxidative stress levels. Previous studies have shown that PAGln can induce endothelial cell activation by increasing oxidative stress, which in turn contributes to endothelial dysfunction [58]. Our results suggest that PAGln exacerbates ischemic brain injury, potentially involving endothelial cell damage and dysfunction.

Our data demonstrates a positive correlation between PAGln and the gut microbiota related to ischemic stroke and T2D. These bacteria were also observed to exhibit a positive association with neutrophils. Previous research has indicated that dysregulation of gut microbiota in patients with arteriosclerotic cerebral small vessel disease independently amplifies the pro-inflammatory characteristics of neutrophils [59]. Our findings indicate that PAGln may serve as a critical bridging molecule linking gut microbiota imbalance to the exacerbation of ischemic brain injury. Meanwhile, our data demonstrate an association between high plasma PAGln levels and a 90 day poor prognosis in patients with stroke, further substantiating the detrimental role of PAGln in the development of stroke. Therefore, it is very necessary to explore feasible interventions to reduce PAGln production. In dietary management, stroke patients, particularly those with T2D, should reduce phenylalanine consumption to decrease production of PAGln. Although PAGln-specific strains were not investigated in our study, probiotic supplementation may decrease the survival of harmful bacteria, potentially reducing PAGln production as a result. Future research may develop engineered enzymes to hydrolyze PAGln, inhibit PAGln production, or antibodies to neutralize PAGln or its receptor.

## Limitations

First, the low NIHSS scores of the included patients restrict the representativeness of this study. Second, the sample size was relatively small, warranting further expansion. Furthermore, confounding factors such as diet, lifestyle, and fecal status should be controlled to improve the persuasiveness of the experimental results. Third, in the animal experiment, we conducted a basic FMT experiment, and the transplanted fecal supernatant contained various microorganisms, including bacteria, fungi, and possibly a specific concentration of PAGln. Therefore, it was challenging to determine which strain played a role, and specific PAGin-producing strains should be supplemented in the future to rule out the presence of PAGln in feces. Fourth, in our study, we identified several genes associated with PAGln production. Future research should focus on engineering bacteria that target these genes to elucidate the causal relationship between bacterial activity and PAGln production. Finally, the molecular pathways by which PAGln promotes the formation of NETs and the relationship between PAGln-related strains and NETs should be further explored.

## Conclusions

Our study revealed that individuals with ischemic stroke and comorbid T2D exhibit disruptions in gut microbiota and elevated plasma PAGln levels. Elevated PAGln may exacerbate brain infarction through NETs formation, systemic inflammation, and oxidative stress. These findings enhance our understanding of the mechanisms involving gut microbial metabolites in the progression of ischemic stroke with comorbid T2D, highlighting PAGln as a potential therapeutic target for this condition. Further studies of specific strains to target PAGln production are needed to improve ischemic stroke with diabetes.

**Clinical Perspectives**

- Type 2 diabetes (T2D) aggravates ischemic stroke. Phenylacetylglutamine (PAGln) is produced by gut microbiota through catabolizing essential amino acid phenylalanine in the distal colon. The association between PAGln and ischemic stroke patients with T2D remains unclear.
- Our study revealed that individuals with ischemic stroke and T2D exhibit disruptions in gut microbiota and elevated plasma PAGln levels. Elevated PAGln may exacerbate brain infarction through neutrophil extracellular trap formation, systemic inflammation, and oxidative stress.
- These findings enhance our understanding of the mechanisms involving gut microbial metabolites in the progression of ischemic stroke with T2D, highlighting PAGln as a potential therapeutic target for this condition.

## Supplementary material

Online supplementary figure S1

Online supplementary figure S2

Uncited online supplementary material

Online supplementary table S1

Online supplementary table S2

Online supplementary table S3

## Data Availability

FASTQ files of the 16S rRNA gene sequencing are available under SRA accession number PRJNA 820272 (https://dataview.ncbi.nlm.nih.gov/object/PRJNA820272). This paper has been posted as a preprint on Research Square with doi: https://doi.org/10.21203/rs.3.rs-1245321/v2 which is available from https://www.researchsquare.com/article/rs-1245321/v2.

## Open Access

This article has been published open access under our Subscribe to Open programme, made possible through the support of our subscribing institutions, learn more here: https://portlandpress.com/pages/open_access_options_and_prices#conditional

## Abbreviations

AMOVA: Analysis of molecular variance
AUC: area under the ROC curve
CI: confidence interval
ECA: external carotid artery
ELISA: enzyme-linked immunosorbent assays
FMT: fecal microbiota transplantation
GSH-PX: glutathione peroxidase
H3Cit: citrullinated histone H3
HbA1c: glycosylated hemoglobin
KO: KEGG orthology
LEfSe: linear discriminant analysis effect size
MCAO: middle cerebral artery occlusion
MDA: malondialdehyde
NETs: neutrophil extracellular traps
NIHSS: National Institutes of Health Stroke Scale
NS: normal saline
PAGln: phenylacetylglutamine
PCoA: principle co-ordinates analysis
PD: phylogenetic diversity
PPDC: phenylpyruvate decarboxylase
PPFOR: phenylpyruvate:ferredoxin oxidoreductase
QIIME: Quantitative Insights into Microbial Eecology
ROC: receiver operating characteristic
SEM: standard error of mean
T2D: type 2 diabetes
T-SOD: total superoxide dismutase
mRS: Modified Rankin Scale
