# Correction: Elevated phenylacetylglutamine caused by gut dysbiosis associated with type 2 diabetes increases neutrophil extracellular traps formation and exacerbates brain infarction

**DOI:** 10.1042/CS20242943_COR

**Published:** 2025-09-04

The authors of the original article “Elevated phenylacetylglutamine caused by gut dysbiosis associated with type 2 diabetes increases neutrophil extracellular traps formation and exacerbates brain infarction” DOI: 10.1042/CS20242943 would like to make the following corrections:

- Corrections to the r and P values in the description listed on Page 724, paragraph 2.
- The titles of the XY axes of Figures 2C and 2D were incorrectly swapped, and these have now been corrected.
- The X and Y axes titles in Figures 2G and 2H were incorrect. For Figure 2G, these have been changed to Y-axis: “plasma NETs” and X-axis: “NIHSS score upon admission”. For Figure 2H, these have been changed to Y-axis: “plasma NETs” and X-axis: “90 day mRS score”.
- The NETs data in Figure 2E has been updated with the correct dilution factor, and the corresponding data corrected accordingly (Figures 2F-H).

The authors explained that for Figure 2E, when measuring the H3Cit (NETs) concentration using the ELISA kit, the plasma samples were diluted 5-fold before detection. However, an incorrect dilution factor was used during final calculations – during re-checking of the data, ‘5 times diluted’ was mistaken for ‘diluted by 1:5’, and so calculations were made by multiplying by 6. This resulted in uniform inflation of all NETs values. As this was a uniform scaling error affecting all data points equally, correcting the data did not change any statistical results related to NETs. The P-value for the difference in NETs between the two groups remains the same as before the correction. The description and labelling errors were accidentally made due to miswriting.

The requested corrections have been assessed and agreed to by the Editorial Board. The authors apologise for any inconvenience caused and declare that these corrections do not change the results or conclusions of their paper.

The corrected version of Figure 2 is presented here.

**Figure:**
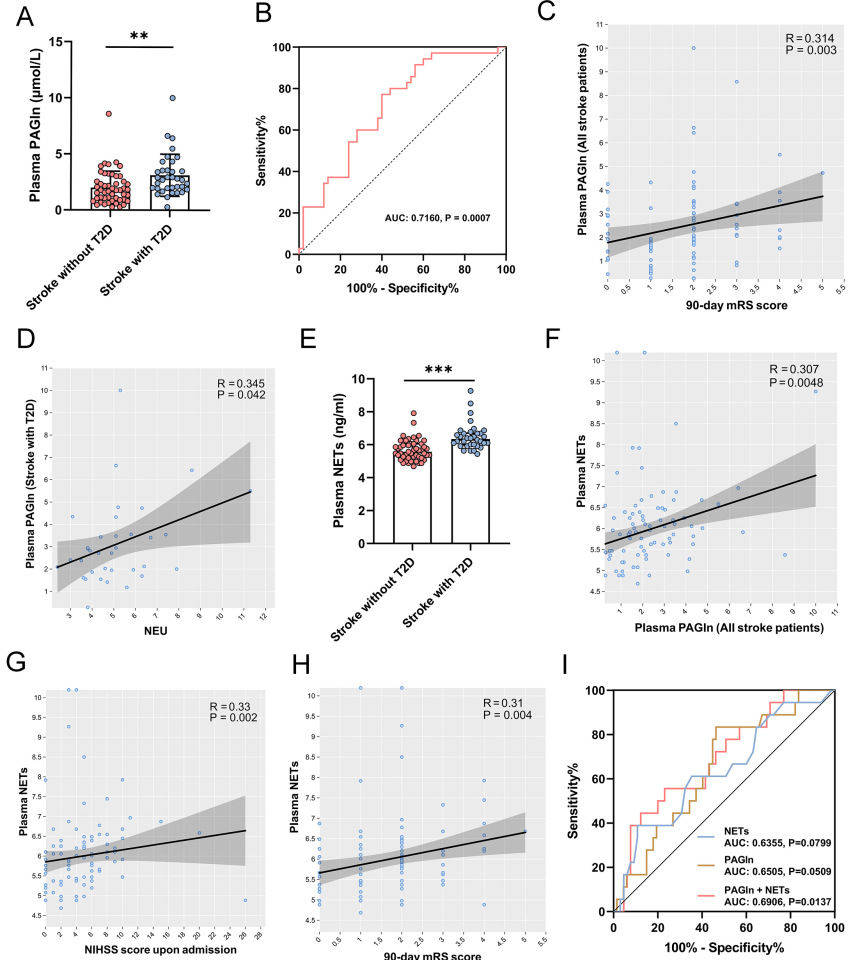


The corrected text can be seen below.

**Results: Gut microbiota-derived PAGln is elevated in plasma of stroke patients with T2D and positively correlated with NETs section**

**Original text:** Spearman correlation analysis revealed a significant correlation between PAGln and H3Cit levels (*r* = 0.41, *P*<0.001)

**Corrected text:** Spearman correlation analysis revealed a significant correlation between PAGln and H3Cit levels (*r* = 0.307, *P*=0.0048; Figure 2F).

