# Supplementary figures and images for: Elevated phenylacetylglutamine caused by gut dysbiosis associated with type 2 diabetes increases neutrophil extracellular traps formation and exacerbates brain infarction
Source: Clin Sci (Lond). 2025 Jun 23;139(12):717–36. doi: 10.1042/CS20242943 (PMC12599254; doi:10.1042/CS20242943)

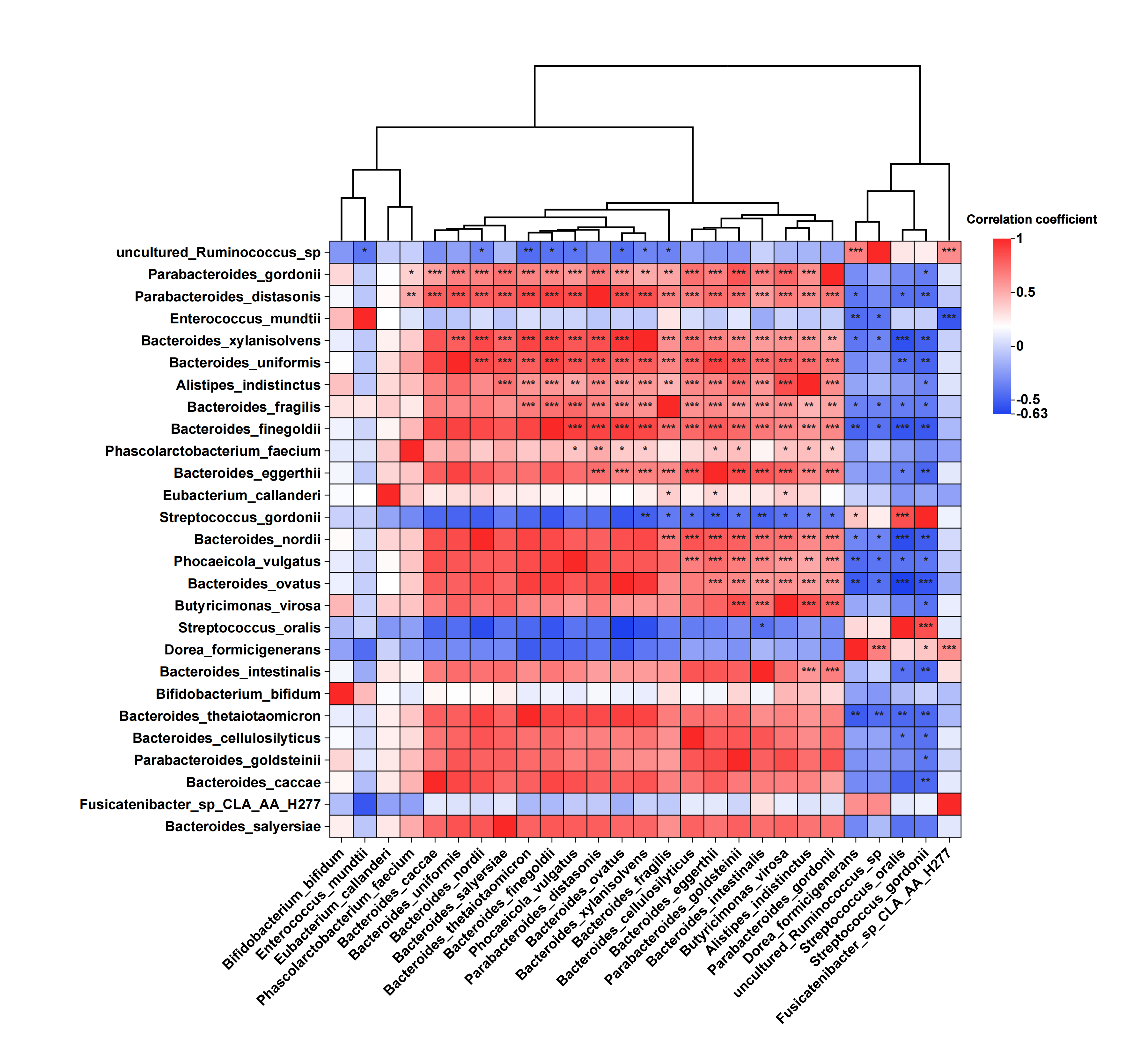

Supplement: Online supplementary figure S1 [file cs-139-12-CS20242943-s001.png]

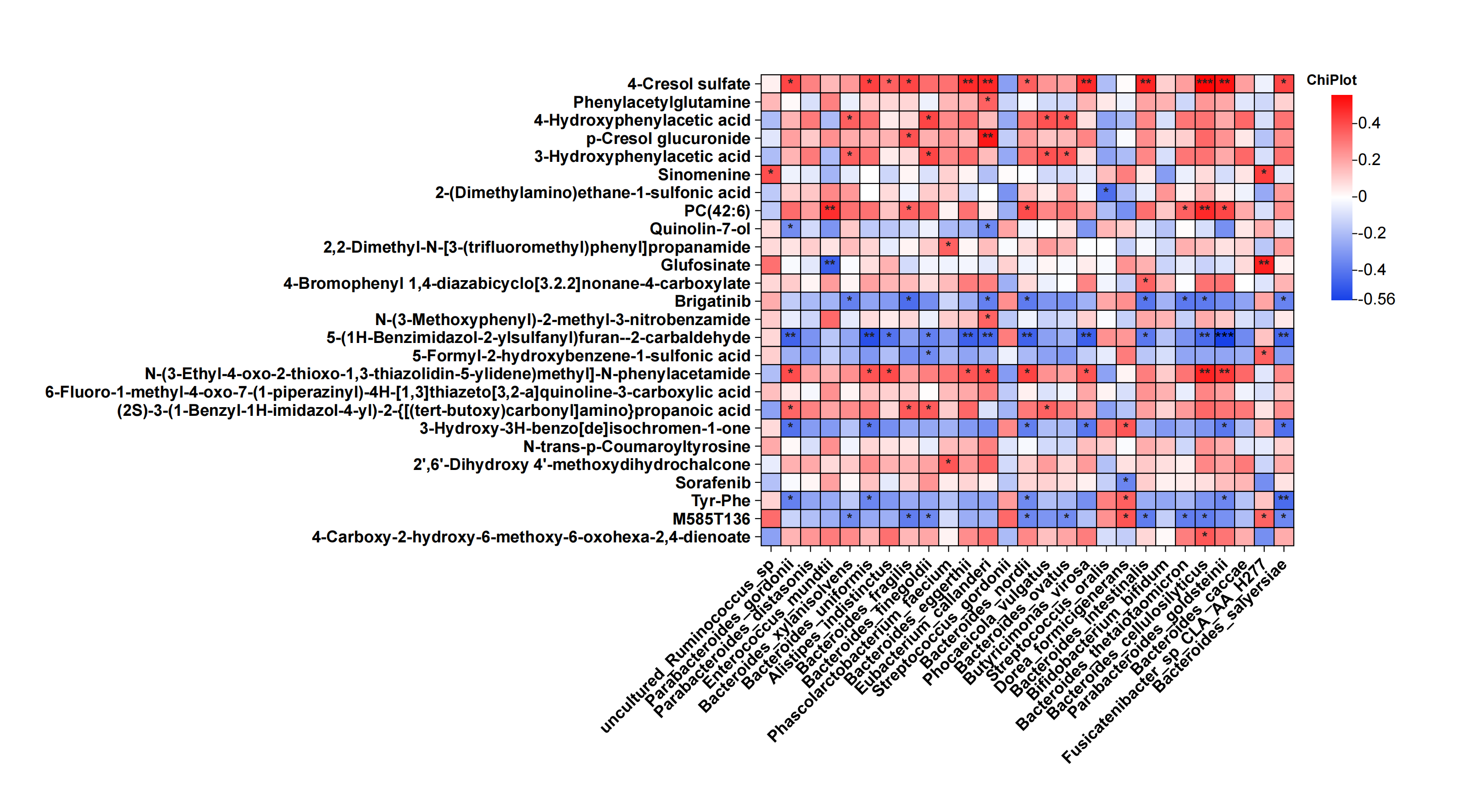

Supplement: Online supplementary figure S2 [file cs-139-12-CS20242943-s002.png]
