# Supplementary material for: Elevated phenylacetylglutamine caused by gut dysbiosis associated with type 2 diabetes increases neutrophil extracellular traps formation and exacerbates brain infarction
Source: Clin Sci (Lond). 2025 Jun 23;139(12):717–36. doi: 10.1042/CS20242943 (PMC12599254; doi:10.1042/CS20242943)
Supplement: Uncited online supplementary material [file cs-139-12-CS20242943-s003.docx]

**Supplemental Fig. 1. The complexity of gut microbial symbiotic network was lower in stroke patients with T2D compared to stroke patients without T2D.** Spearman correlation analysis of differential gut microbiota in two groups. ^*^*P* < 0.05, ^**^*P* < 0.01, ^***^*P* < 0.001.

**Supplemental Fig. 2. Differential plasma metabolites were correlated with differential gut microbiota.** Spearman correlation analysis of differential plasma metabolites and differential gut microbiota. ^*^*P* < 0.05, ^**^*P* < 0.01, ^***^*P* < 0.001.
