# Supplementary material for: Elevated phenylacetylglutamine caused by gut dysbiosis associated with type 2 diabetes increases neutrophil extracellular traps formation and exacerbates brain infarction
Source: Clin Sci (Lond). 2025 Jun 23;139(12):717–36. doi: 10.1042/CS20242943 (PMC12599254; doi:10.1042/CS20242943)
Supplement: Online supplementary table S1 [file cs-139-12-CS20242943-s004.docx]

**Supplemental Table 1**

Characteristics of clinical cohort 1

| Baseline characteristics | Stroke without T2D (n=50) | Stroke with T2D (n=35) | *P-*value |
| --- | --- | --- | --- |
| Sex (male/female) | 31/19 | 24/11 | 0.533 |
| Age (year) | 57.62 ± 12.08 | 60.14 ± 11.95 | 0.344 |
| Hypertension | 36 (72) | 31 (89) | 0.066 |
| Dyslipidemia | 13 (26) | 13 (37) | 0.273 |
| Coronary heart disease | 5 (10) | 6 (17) | 0.334 |
| Current smoking | 26 (52) | 16 (46) | 0.568 |
| Admission NIHSS score | 3 (1, 6) | 6 (4, 8) | 0.002 |
| 90-day mRS score | 1 (1, 2) | 2 (2, 3) | 0.003 |
| White blood cells (×10^9^/µL) | 6.5 (5.3, 8.2) | 7.3 (6.2, 8.8) | 0.165 |
| Neutrophils (×10^9^/µL) | 4.4 (3.4, 4.9) | 5.0 (3.8, 6.0) | 0.045 |
| Lymphocytes (×10^9^/µL) | 1.4 (1.1, 2.1) | 1.6 (1.2, 2.0) | 0.69 |
| NLR | 2.4 (2.0, 3.8) | 3.3 (2.3, 4.4) | 0.189 |
| Blood urea nitrogen (mM) | 4.64 (3.94, 5.93) | 5.35 (4.45, 6.54) | 0.097 |
| Serum creatinine (mM) | 78.0 (65.4, 78.6) | 80.1 (71.0, 93.7) | 0.841 |
| Triglycerides (mM) | 1.47 (1.06, 2.22) | 1.54 (1.26, 2.04) | 0.556 |
| Total cholesterol (mM) | 4.22 (3.38, 4.92) | 3.55 (3.06, 5.02) | 0.296 |
| Low-density lipoprotein (mM) | 2.68 (1.99, 3.15) | 2.23 (1.84, 3.00) | 0.313 |
| Glucose (mM) | 5.23 (4.81, 5.67) | 9.20 (6.24, 10.82) | <0.001 |
| HbA1c (%) | 5.6 (5.5, 5.9) | 7.8 (6.7, 8.4) | <0.001 |
| Homocysteine (μM) | 12.9 (9.8, 17.8) | 13.9 (10.8, 15.5) | 0.979 |
| Prothrombin time (s) | 12.1 (11.5, 12.8) | 11.9 (11.6, 12.7) | 0.884 |
| Activated partial thromboplastin time (s) | 28.5 (25.9, 30.2) | 27.6 (26.1, 29.5) | 0.535 |
| Fibrinogen (g/L) | 3.05 (2.51, 3.61) | 3.11 (2.62, 3.56) | 0.939 |
| D-dimer (mg/L) | 0.15 (0.10, 0.25) | 0.16 (0.06, 0.20) | 0.841 |

Data are expressed as number (sex), mean ± SEM (age), number (percentage) (risk factors) or median (interquartile range) (clinical findings and biochemical index).

The Student’s t-test or Mann-Whitney U test was used for pairwise comparison.

*P* < 0.05 is considered significant.

NIHSS, National Institutes of Health Stroke Scale; mRS, Modified Rankin Scale; NLR: neutrophil-lymphocyte ratio; HbA1c: glycated hemoglobin.
