# Supplementary material for: Elevated phenylacetylglutamine caused by gut dysbiosis associated with type 2 diabetes increases neutrophil extracellular traps formation and exacerbates brain infarction
Source: Clin Sci (Lond). 2025 Jun 23;139(12):717–36. doi: 10.1042/CS20242943 (PMC12599254; doi:10.1042/CS20242943)
Supplement: Online supplementary table S2 [file cs-139-12-CS20242943-s005.docx]

**Supplemental Table 2**

Characteristics of clinical cohort 2

| Baseline characteristics | Stroke without T2D (n=19) | Stroke with T2D (n=15) | *P-*value |
| --- | --- | --- | --- |
| Sex (male/female) | 9/10 | 10/5 | 0.260 |
| Age (year) | 61.26 ± 11.97 | 63.47 ± 12.49 | 0.605 |
| Hypertension | 11 (58) | 12 (80) | 0.171 |
| Dyslipidemia | 13 (68) | 8 (53) | 0.369 |
| Coronary heart disease | 3 (16) | 0 (0) | 0.107 |
| Current smoking | 10 (53) | 6 (40) | 0.464 |
| Admission NIHSS score | 2 (1, 5) | 2 (1, 7) | 0.242 |
| 90-day mRS score | 1 (0, 1) | 2 (1, 2) | 0.06 |
| White blood cells (×10^9^/µL) | 6.6 (5, 9.1) | 6.2 (5.4, 7.4) | 0.837 |
| Neutrophils (×10^9^/µL) | 4.5 (3.4, 7.9) | 3.5 (3, 5.4) | 0.336 |
| Lymphocytes (×10^9^/µL) | 1.4 (1.1, 1.6) | 1.6 (1.1, 2) | 0.336 |
| NLR | 2.93 (2.06, 7.45) | 2.78 (2.19, 3.57) | 0.56 |
| Blood urea nitrogen (mM) | 5.21 (4.55, 6.39) | 5.5 (4.32, 7.12) | 0.681 |
| Serum creatinine (mM) | 63 (55.7, 72) | 73 (57.2, 112) | 0.179 |
| Triglycerides (mM) | 1.57 (1.33, 2.17) | 1.25 (0.99, 1.74) | 0.228 |
| Total cholesterol (mM) | 4.48 (4.02, 5.39) | 4.22 (3.34, 5.33) | 0.271 |
| Low-density lipoprotein (mM) | 2.82 (2.52, 3.47) | 2.66 (1.92, 3.21) | 0.242 |
| Glucose (mM) | 6.52 (5.39, 7.34) | 6.81 (5.28, 9.45) | 0.451 |
| HbA1c (%) | 5.8 (5.6, 6.1) | 6.1 (5.9, 8.6) | 0.019 |
| Homocysteine (μM) | 13.35 (10.16, 15.7) | 13.89 (11.07, 15.15) | 0.493 |
| Prothrombin time (s) | 11.2 (10.9, 12.5) | 11.3 (10.5, 11.6) | 0.336 |
| Activated partial thromboplastin time (s) | 25.8 (24.5, 26.9) | 25.1 (23.7, 26) | 0.242 |
| Fibrinogen (g/L) | 3.02 (2.35, 3.33) | 3.1 (2.78, 4.38) | 0.354 |
| D-dimer (mg/L) | 0.19 (0.07, 0.36) | 0.17 (0.09, 0.28) | 0.837 |

Data are expressed as number (sex), mean ± SEM (age), number (percentage) (risk factors) or median (interquartile range) (clinical findings and biochemical index).

The Student’s t-test or Mann-Whitney U test was used for pairwise comparison.

*P* < 0.05 is considered significant.

NIHSS, National Institutes of Health Stroke Scale; mRS, Modified Rankin Scale; NLR: neutrophil-lymphocyte ratio; HbA1c: glycated hemoglobin.
