# Supplementary material for: Elevated phenylacetylglutamine caused by gut dysbiosis associated with type 2 diabetes increases neutrophil extracellular traps formation and exacerbates brain infarction
Source: Clin Sci (Lond). 2025 Jun 23;139(12):717–36. doi: 10.1042/CS20242943 (PMC12599254; doi:10.1042/CS20242943)
Supplement: Online supplementary table S3 [file cs-139-12-CS20242943-s006.docx]

**Additional Table 2**

Characteristics of donors of fecal microbiota transplants.

| Baseline characteristics | Stroke without T2D (n=5) | Stroke with T2D (n=5) | *P-*value |
| --- | --- | --- | --- |
| Gender (male/female) | 4/1 | 5/0 | 0.292 |
| Age (year) | 64.20 ± 7.05 | 62.20 ± 10.83 | 0.841 |
| Hypertension | 3 (60.0) | 5 (100.0) | 0.114 |
| Dyslipidemia | 3 (60.0) | 3 (60.0) | 1.000 |
| Coronary heart disease | 5 (100.0) | 5 (100.0) | / |
| Current smoking | 2 (40.0) | 3 (60.0) | 0.527 |
| Admission NIHSS score | 6 (4, 9) | 4 (4, 9) | 0.841 |
| 90-day mRS score | 1 (1, 2) | 2 (2, 3) | 0.095 |
| White blood cells (×10^9^/µL) | 6.5 (5.7, 8.8) | 6.7 (5.6, 9.5) | 0.841 |
| Neutrophils (×10^9^/µL) | 4.4 (4.25, 7.5) | 5.2 (3.7, 6.95) | 0.841 |
| Lymphocytes (×10^9^/µL) | 1 (0.45, 1.25) | 1.3 (1.1, 1.65) | 0.950 |
| NLR | 6.17 (3.79, 16.18) | 3.38 (2.91, 5.123) | 0.950 |
| Blood urea nitrogen (mM) | 4.37 (4.03, 6.56) | 6.49 (5.80, 9.53) | 0.222 |
| Serum creatinine (mM) | 66 (64.55, 99.00) | 80.2 (55.85, 104.50) | 0.548 |
| Triglycerides (mM) | 1.3 (0.78, 4.04) | 1.45 (1.28, 1.70) | 0.841 |
| Total cholesterol (mM) | 3.64 (2.59, 7.06) | 3.55 (2.80, 3.915) | 0.690 |
| Low-density lipoprotein (mM) | 2.25 (1.53, 4.14) | 2.22 (1.76, 2.67) | 0.841 |
| Glucose (mM) | 6.06 (5.50, 6.31) | 9.2 (6.36, 9.85) | 0.016 |
| HbA1c (%) | 5.8 (5.35, 6.1) | 7.7 (7.25, 8.85) | 0.008 |
| Homocysteine (μM) | 11.43 (8, 17.97) | 15.47 (9.57, 19.17) | 0.690 |
| PAGln (mM) | 1.52 ± 0.37 | 5.41 ± 2.80 | 0.008 |

Data are expressed as number (sex), mean ± SEM (age), number (percentage) (risk factors) or median (interquartile range) (clinical findings and biochemical index).

The Student’s t-test or Mann-Whitney U test was used for pairwise comparison.

*P* < 0.05 is considered significant.

NIHSS, National Institutes of Health Stroke Scale; mRS, Modified Rankin Scale; NLR: neutrophil-lymphocyte ratio; HbA1c: glycated hemoglobin.
